# Insulin-like growth factor 1 supplementation supports motor coordination and affects myelination in preterm pigs

**DOI:** 10.3389/fnins.2023.1205819

**Published:** 2023-06-19

**Authors:** Line I. Christiansen, Gemma C. Ventura, Bo Holmqvist, Karoline Aasmul-Olsen, Sandy E. H. Lindholm, Matthew D. Lycas, Yuki Mori, Jan Bojsen-Møller Secher, Douglas G. Burrin, Thomas Thymann, Per T. Sangild, Stanislava Pankratova

**Affiliations:** ^1^Comparative Pediatrics and Nutrition, Department of Veterinary and Animals Sciences, Faculty of Health and Medical Sciences, University of Copenhagen, Frederiksberg, Denmark; ^2^ImaGene-iT AB, Lund, Sweden; ^3^Department of Neuroscience, Faculty of Health and Medical Sciences, University of Copenhagen, Copenhagen, Denmark; ^4^Center for Translational Neuromedicine, Faculty of Health and Medical Sciences, University of Copenhagen, Copenhagen, Denmark; ^5^Department of Veterinary Clinical Sciences, Faculty of Health and Medical Sciences, University of Copenhagen, Frederiksberg, Denmark; ^6^United States Department of Agriculture, Agricultural Research Service Children’s Nutrition Research Center, Department of Pediatrics, Baylor College of Medicine, Houston, TX, United States; ^7^Department of Neonatology, Rigshospitalet, Copenhagen, Denmark; ^8^Department of Pediatrics, Odense University Hospital, Odense, Denmark; ^9^Faculty of Theology, University of Copenhagen, Copenhagen, Denmark

**Keywords:** IGF-1, motor function, preterm neonates, myelination, caudate nucleus, hippocampus

## Abstract

**Introduction:**

Preterm infants have increased risk of impaired neurodevelopment to which reduced systemic levels of insulin-like growth factor 1 (IGF-1) in the weeks after birth may play a role. Hence, we hypothesized that postnatal IGF-1 supplementation would improve brain development in preterm pigs, used as a model for preterm infants.

**Methods:**

Preterm pigs delivered by cesarean section received recombinant human IGF-1/IGF binding protein-3 complex (rhIGF-1/rhIGFBP-3, 2.25 mg/kg/day) or vehicle from birth to postnatal day 19. Motor function and cognition were assessed by monitoring of in-cage and open field activities, balance beam test, gait parameters, novel object recognition and operant conditioning tests. Collected brains were subject to magnetic resonance imaging (MRI), immunohistochemistry, gene expression analyses and protein synthesis measurements.

**Results:**

The IGF-1 treatment increased cerebellar protein synthesis rates (both *in vivo* and *ex vivo*). Performance in the balance beam test was improved by IGF-1 but not in other neurofunctional tests. The treatment decreased total and relative caudate nucleus weights, without any effects to total brain weight or grey/white matter volumes. Supplementation with IGF-1 reduced myelination in caudate nucleus, cerebellum, and white matter regions and decreased hilar synapse formation, without effects to oligodendrocyte maturation or neuron differentiation. Gene expression analyses indicated enhanced maturation of the GABAergic system in the caudate nucleus (decreased *NKCC1:KCC2* ratio) with limited effects in cerebellum or hippocampus.

**Conclusion:**

Supplemental IGF-1 during the first three weeks after preterm birth may support motor function by enhancing GABAergic maturation in the caudate nucleus, despite reduced myelination. Supplemental IGF-1 may support postnatal brain development in preterm infants, but more studies are required to identify optimal treatment regimens for subgroups of very or extremely preterm infants.

## Introduction

Premature birth abruptly ends an important period of intrauterine maturation for the brain and predisposes the infants to immediate and future adverse neurodevelopmental outcomes (Ream and Lehwald, 2017). Every year around 15 million infants worldwide are born prematurely (< 37 weeks gestation), and with advancements in neonatal care, this population is becoming bigger and with ever decreasing gestational age at birth (Blencowe et al., 2012; Chawanpaiboon et al., 2019). Finding an effective and safe intervention to support brain development for premature infants is therefore needed.

An important factor for fetal and postnatal organ development, including brain growth and maturation, is local insulin-like growth factor 1 (IGF-1) production (O’Kusky and Ye, 2012; Hellström et al., 2016). The serum concentration of IGF-1 peaks during the third trimester, decreases after birth, and then gradually increases postnatally (Bang et al., 1994; Juul et al., 1995). However, the period of low IGF-1 levels following birth is prolonged for infants born very preterm (< 32 weeks gestation) compared with infants born at term (Lineham et al., 1986; Hellström et al., 2016). Levels of systemic IGF-1 are positively associated with head circumference, brain volume, white and grey matter volumes, and neurodevelopmental outcomes at 2 years of age (Löfqvist et al., 2006; Hansen-Pupp et al., 2011, 2013; Hellström et al., 2022). Recently, intravenous supplementation with a complex of recombinant human (rh)IGF-1 and IGF binding protein-3 (rhIGF-1/rhIGFBP-3) to hospitalized preterm infants indicated benefits to decrease the incidence of bronchopulmonary dysplasia (BPD) and intraventricular hemorrhage (IVH) (Ley et al., 2019; Horsch et al., 2020).

IGF-1 is expressed throughout the brain but most endogenous IGF-1 is synthesized and secreted by the liver before entering the systemic circulation, where it can cross into the central nervous system *via* the choroid plexus (Carro et al., 2000; Nishijima et al., 2010). The bioavailability of IGF-1 is regulated by six IGF binding proteins (IGFBPs1-6) where 80% of the circulating peptide amount is bound to IGFBP-3 (Rajaram et al., 1997). IGF-1 mediates signaling through the insulin receptor and its specific IGF-1 receptor (IGF1R) which is expressed throughout the brain by several types of cells and controls neurodevelopmental processes including synapse formation, myelination, and plasticity (Marks et al., 1991; Garcia-Segura et al., 1997; Zeger et al., 2007; Suh et al., 2013; Ziegler et al., 2015). The importance of IGF-1 for brain development is evident from transgenic animal studies showing that IGF-1 loss in the brain resulted in microcephaly and hypomyelination, while overexpression of IGF-1 led to increased brain volume (Carson et al., 1993; Beck et al., 1995). Infant IGF-1 gene defects are associated with microcephaly and growth failure (Netchine et al., 2011) but cause-effect relationships among IGF-1 levels, growth failure and impaired brain development in preterm infants remains unknown.

**Figure 1:**
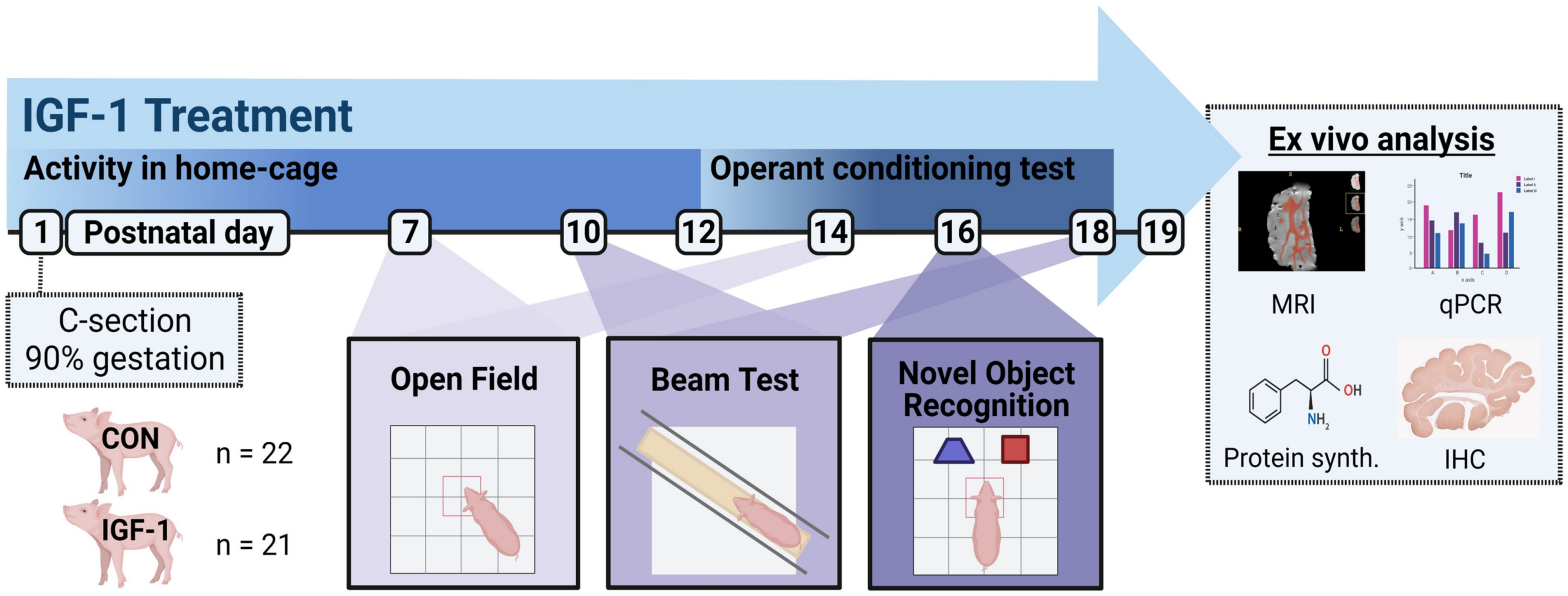
Timeline of the study design. Pigs were born preterm by cesarian section at 90% of gestation. The pigs were stratified according to birth weight and sex into two groups: Control (CON) receiving vehicle treatment (*n* = 22) and IGF-1 receiving rhIGF-1/rhIGFBP3 (*n* = 21) throughout the study period. Activity in the home-cage was measured from PND1-12, and from PND12-18 an operant conditioning test was performed 7 times daily in the home-cages. Open field test was performed on PND 7 and 14, balance beam test was performed on PND10 and 18, and novel object recognition was performed on PND16. Tissues were collected on PND19 for analysis of MRI, qPCR, protein synthesis, and IHC. Created with BioRender.com.

Similar to preterm infants, preterm pigs have lower systemic levels of IGF-1 for a long period after birth compared with term controls (Andersen et al., 2016; Holgersen et al., 2021). We recently showed that IGF1R is highly expressed in the preterm pig cerebral cortex, periventricular white mater (PvWM) and hippocampus, and in close association with immature neurons (Christiansen et al., 2023). Furthermore, we showed that preterm pigs supplemented with IGF-1 for 5–9 days have enhanced hippocampal neuronal differentiation and increased subcortical myelination, suggesting that IGF-1 promotes both grey and white mater maturation (Christiansen et al., 2023). It is important to know safety and efficacy if supplementation is continued in the following weeks when preterm pigs start to become fully mobile and able to live independently without intensive care support and artificial feeding (Sangild et al., 2013; Andersen et al., 2016; Bæk et al., 2021). As brain development is a continuous process composed of several overlapping events with regional-dependent timing, continued exposure to supplemental IGF-1 may act differently than short-term exposure just after birth (Andersen, 2003). We hypothesized that prolonged IGF-1 supplementation would improve neurodevelopmental outcomes for preterm pigs, beyond the immediate neonatal period into the anabolic growth phase. We explored our hypothesis by supplementing preterm pigs with IGF-1/IGFBP-3 for 19 days, aiming to reach plasma levels reflecting those in term pigs of similar age (Holgersen et al., 2021). This extended treatment period after preterm birth allowed us to investigate structural brain outcomes but also aspects related to brain function, such as cognition and motor development (Figure 1).

## Methods

### Animal model

The study was approved by the Danish National Committee on Animal Experimentation (license no.: 2020-15-0201-00520) and complied with ARRIVE guidelines.

Preterm pigs were delivered by cesarean section at 90% gestation (gestational age 106 days, *n* = 43, male/female = 24/19, from two sows of Danish Landrace × Yorkshire × Duroc crossbreed). Following delivery, all pigs were resuscitated and fitted with an orogastric feeding tube and an umbilical catheter. The pigs were stratified according to birth weight and sex and randomly allocated into two groups: one treated with rhIGF-1/rhIGFBP-1 complex (2.25 mg/kg/day; mecasermin rinfabate; Takeda, Cambridge, MA, USA) dissolved in formulation buffer as vehicle (50 nM sodium acetate, 105 mM sodium chloride, 0.005% (v/v) polysorbate 20, pH 5.5), hereafter named “IGF-1 group,” and the other group of pigs treated with vehicle (‘Control’ group). Treatment was administered by continuous intraarterial infusion on postnatal day one to eight (PND1-8) and then by three daily injections *via* a subcutaneous catheter on PND9-19. Throughout the study, the pigs were housed individually in temperature regulated environments and on PND1-5 in oxygenated incubators. The pigs were fed every 2 h during the day and every 3 h during the night on PND1-9, from PND10 they were fed every 3 h throughout the day. The diet consisted of increasing enteral volumes of raw bovine milk (32–180 mL/kg/day) supplemented until PND8 with parental nutrition in decreasing volumes (120–48 mL/kg/day). The raw bovine milk used for enteral nutrition was supplemented with 7 g/L vitamins (Phlexy-Vits; Nutricia, Zoetermeer, Netherlands), 25 g/L whey proteins (DI9025; Arla Foods Ingredients, Viby, Denmark) and 6 g/L electrolytes (Revolyt Nutrition; Gunnar Kjems, Copenhagen, Denmark), while the parental nutrition was based on the commercially available product (Kabiven; Fresenius Kabi, Uppsala, Sweden), modified as previously described (Sangild et al., 2013). Body weight of the pigs was recorded daily, and they were clinically assessed twice daily as previously described (Holme Nielsen et al., 2019). Within the first 48 h of life, all pigs were infused with sterile maternal sow plasma *via* the umbilical catheter (25 mL/kg) to provide passive immunity. All pigs were treated prophylactically with a combination of oral antibiotics: Amoxicillin/clavulanic acid (2care4, Esbjerg, Denmark), gentamicin (Gentocin Vet; Scanvet, Fredensborg, Denmark), metronidazole (Flagyl; Sanofi A/S, Paris, France) from PND8-10. On PND19, the pigs were anesthetized and then euthanized with an intracardiac injection of pentobarbital sodium (Euthanimal Vet; Alfasan, Woerden, The Netherlands). The total brain and selected regions were dissected, weighed and snap-frozen or fixed in paraformaldehyde, as described previously (Christiansen et al., 2023).

### Behavioral analysis

All parts of the *in vivo* study, including treatment administration, scoring, behavioral tests, and analyses were performed by blinded investigators. Animals who were immobile, had severe diarrhea, were severely dehydrated, emaciated, or fatigued and non-responsive, were omitted from behavioral tests and in the most severe cases, euthanized prior to assessment.

#### Acquisition of basic motor functions

The acquisition of neuromuscular parameters (first eyelid opening, first stand, first walk, first drinking from a trough) was recorded individually for each animal, as previously described (Cao et al., 2015). Individual motor activity in the home-cage was recorded continuously from 12 h after the cesarean section (PND1) until PND10, using a motion-sensitive infrared surveillance camera. The activity level was estimated using the PIGLWin software program (Ellegaard Systems, Faaborg, Denmark). A filter was applied to the data to sort out activity shorter than 5 s, thereby excluding minor spontaneous movements, such as leg twitches.

#### Open field

Locomotor activity was evaluated on PND7 and PND14 in a 2 × 2 m open field with black wooden walls and rubber flooring, essentially using the method described previously (Holme Nielsen et al., 2019). Recorded videos were analyzed with the video tracking software EthoVision XT10 (Noldus Information Technology, Wageningen, The Netherlands) (Andersen et al., 2016). Of the 37 viable pigs on PND7, 33 pigs were included (Control: *n* = 18, m/*f* = 9/9. IGF-1: *n* = 15, m/*f* = 8/7), and 4 pigs (IGF-1) were excluded (2 had severe diarrhea, 1 was fatigued and non-responsive, 1 had severely distended abdomen). Of the 31 viable pigs on PND14, 29 pigs were included (Control: *n* = 13, m/*f* = 7/6. IGF-1: *n* = 16, m/f = 9/7), and 2 pigs (1 Control, 1 IGF-1) were excluded (both immobile).

#### Balance beam test and gait analysis

Balance and coordination of the animals was assessed on PND10 and PND18 as previously described (Roelofs et al., 2019; Henriksen et al., 2022b). The animals completed three consecutive runs along a 180 cm long and 4.5 cm high wooden beam and the number of mis-steps down from the beam were counted. The width of the beam was adjusted according to the shoulder width of the pig (approximately 4 cm wider). Mis-steps were counted based on 4 criteria: (1) Only during forward motion; (2) simultaneous mis-steps with several feet counted as one; (3) all feet should be on the beam prior to mis-stepping; (4) foot must touch the floor. Transverse plane video recordings of the trials were used for analysis of gait parameters as previously described (Henriksen et al., 2022b). Briefly, tracking of the pigs was performed using DeepLabCut (version 2.0.7.2) (Nath et al., 2019), where a model was trained to identify the snout of the pigs, the front shoulder, the tail base, and each independent foot. Data gathered from the model were subjected to computerized program script (Python) to generate measurements of stride length, swing time, and stance time of the longest period of continuous motion of the pigs. These measurements were normalized as previously described (Holme Nielsen et al., 2019). The code for video analysis can be found at https://github.com/Comparative-Pediatrics-and-Nutrition/.

Of the 33 viable pigs on PND10, 28 pigs were included (Control: *n* = 14, m/*f* = 6/8. IGF-1: *n* = 15, m/*f* = 8/7), and 3 pigs (2 Control, 1 IGF-1) were excluded (2 were immobile, 1 did not complete the test). Of the 30 viable pigs on PND18, 27 pigs were included (Control: *n* = 12, m/*f* = 7/5. IGF-1: *n* = 15, m/*f* = 9/6), and 3 pigs (1 Control, 2 IGF-1) were excluded (2 were immobile, 1 due to malfunctioning video recording).

#### Operant conditioning test

Starting on PND12 and continuing until PND19, the pigs were assessed seven times daily by an in-cage operant conditioning test (NorthTech, Copenhagen, Denmark). The test is based on classical operant behavior and quantifies the associative learning capabilities of the animals (Kornum and Knudsen, 2011). A panel with two blue light cues was installed on the inside of the cage door. During testing periods, one of the two cues will light up, alternating randomly between the left and right cue. Each testing period, which is 1 h before feeding, was initiated by a sound to ensure that the animals were awake and motivated for the task. When the animal touched the lightened cue, a 1 mL chocolate milk reward was released into their trough. Conversely, touching the non-lighted cue elicited a 60 s penalty where both lights were turned off (i.e., no possibility to obtain milk reward following an incorrect response). Each right and wrong answer (max 10) during the 1 h test periods was recorded. The animals had no learning period prior to testing and no habituation as it was performed in their individual home cages. To avoid overfeeding the animals, the test was terminated for animals who reached the learning criteria: answering eight out of ten tasks correctly in two subsequent trials. The reward bottle, tube and pump were rinsed/refilled daily prior to testing.

Of the 30 viable pigs on PND12, 23 pigs were included (Control: *n* = 11, m/*f* = 7/4. IGF-1: *n* = 12, m/*f* = 6/6), and 8 pigs (3 Control, 5 IGF-1) were excluded (7 displayed continuous off-task behavior: <10% participation throughout the test period, 1 was immobile).

#### Novel object recognition

On PND16 the animals were tested in a novel object recognition setup as previously described to evaluate short-term memory and exploratory behavior (Henriksen et al., 2021). In short, the trial consisted of three phases each taking 5 min: Familiarization phase, resting phase, and a test phase. The animals were previously habituated to the environment on PND7 and PND14 during the open field recordings. In the familiarization phase, the animals were exposed to two identical objects (white plastic containers 29 × 18 × 13 cm) not previously encountered. In the resting phase, the animals were placed back in their home-cage while one of the objects in the arena was replaced at random with a novel object (blue kettlebell 21 × 17 × 10 cm). In the test phase, the animals were again placed in the center of the arena and the amount of time exploring the two different objects were measured. The familiarization and trial phase were recorded from a dorsal plane, and the videos were analyzed using DeepLabCut (version 2.0.7.2) (Nath et al., 2019). The model was trained to detect the snout of the pig, the sides of its head, as well as the front and back sides of the body. The novel objects were segmented from the image by hand. The time spent investigating (snout contact) the novel object relative to the total time spent investigating both objects in the test phase (recognition index) was estimated by identifying the time in the video where the coordinates for the detected head of the pig was in contact with the segmented area of the object.

Of the 30 viable pigs on PND16, 27 pigs were included (Control: *n* = 12, m/*f* = 7/5. IGF-1: *n* = 16, m/*f* = 9/7), and 3 pigs (2 Control, 1 IGF-1) were excluded (2 displayed continuous off-task behavior: total time spent investigating objects was <2 s, 1 was immobile).

### Magnetic resonance imaging

The volume of the right brain hemisphere, white matter, and grey matter were measured by MRI as previously described (Henriksen et al., 2022a). Briefly, the entire right hemisphere was immersed in 4% paraformaldehyde in phosphate buffered saline for 24 h at 4°C (PBS; 0.1 M, pH 7.4), rehydrated with PBS for 24 h at 4°C and washed twice with Fomblin (perfluoro-polyether; Solvay, Princeton, New Jersey, USA) to remove the excess fluid and resubmerged in clean Fomblin prior to scanning. It was confirmed that there was no surrounding fluid signal on the surface of each brain before high-resolution imaging. Data was acquired with a 9.4 Tesla preclinical scanner (Bruker BioSpin, Ettlingen, Germany) equipped with a 240 mT/m gradient coil (BGA-12S, Bruker) and using an 86-mm inner diameter transmit-receive volume coil. The imaging protocol used a 3D gradient-spoiled steady state free precession, and the parameters were set to: repetition time = 4.6 ms, echo time = 2.3 ms, number of signals averaged = 10, flip angle = 25°, field of view = 60 mm × 38.4 mm × 25.6 mm, matrix = 300 × 192 × 128, image resolution = 200 μm isotropic, acquisition time = 20 min. The images were bias-field corrected, and segmented as previously described (Henriksen et al., 2022a). The volume of white and grey matter was normalized to the total volume of the brain hemisphere.

### Brain protein synthesis

The protein synthesis rate in the cerebellum was measured both *in vivo* and *ex vivo*. The *in vivo* measurement was performed as previously described (Rudar et al., 2021; Christiansen et al., 2023). In brief, animals were injected with a dose (10 mL/kg) of L-phenylalanine (Phe; 1.5 mmol/kg) containing L-[ring-^2^H_5_]Phe at 30 mol % (0.45 mmol/kg; Cambridge Isotope Laboratories, Tewksbury, MA, USA) 30 min prior to euthanasia. Cerebellar tissue was collected and immediately snap-frozen in liquid nitrogen. The tissue was analyzed for tracer accumulation and the fractional rates of protein synthesis (Ks; protein mass synthesized/day) was calculated.

The protein synthesis rate was validated *ex vivo* utilizing the principles of surface sensing translation (SUnSET) and western blot as described previously (Schmidt et al., 2009; Goodman et al., 2011). Following dissection of cerebellar tissue, the samples were preincubated for 15 min in tissue buffer (Hibernate-A, Gibco, Waltham, MA, USA), then treated 30 min in 1 μM puromycin (Sigma-Aldrich, St. Louis, MO, USA) diluted in tissue buffer and immediately snap-frozen in liquid nitrogen. The frozen samples were disrupted with a pestle on ice and lyzed with neuronal protein extraction reagent (N-PER) supplemented with HALT Protease Inhibitor Cocktail kit and 0.5 M EDTA solution (all from Thermo Fisher Scientific, Waltham, MA, USA). Protein concentration was determined using Pierce BCA Protein Assay kit (Thermo Fisher Scientific) according to manufacturer’s protocol. Protein extracts (30 μg) were loaded onto Mini-PROTEAN TGX Precast Gels (Bio-Rad Laboratories, Hercules, CA, USA). The gels were electro-transferred to 0.2 μm PVDF membranes (Bio-Rad Laboratories). The blots were subsequently blocked in 5% milk in PBS, 0.05% Tween 20 for 1.5 h at room temperature and then incubated at 4°C overnight with IgG2ak monoclonal anti-puromycin antibody (1:2000, MABE343, Merck Millipore, Burlington, MA, USA). Bands were detected with horseradish peroxidase-conjugated anti-mouse IgG Fc 2a antibody (1:5000, 115–035-164, Jackson Immuno Research Labs, West Grove, PA, USA), developed with SuperSignal TMB reagent (Thermo Fisher Scientific) and visualized with an Amersham ImageQuant 800 imaging system (Cytiva Europe GmbH, Germany). Band intensity was quantified using ImageJ program (version 10.2, NIH, Berthesda, MD, USA).

### Immunohistochemistry and image data processing

IHC analysis of brain tissue was performed as previously described (Christiansen et al., 2023). Briefly, the entire right brain hemisphere was stored in 4% paraformaldehyde in PBS for collectively 72 h, cut in 7–8 mm tissue slabs, embedded in paraffin and the same brain region of all samples was cut in coronal sections (5 μm), at two levels of selected regions (at least 30 μm apart). Sections were collected on SuperFrost Plus microscope slides and were deparaffinized and rehydrated. Sections were then processed for heat induced epitope retrieval (HIER), using Tris-EDTA pH 9.0 (Olig-2) or Citrate buffer, pH 6.0 (all other epitopes), around 5 min with end-temperatures at 95°C. Hematoxylin–eosin (HE) staining was made to ensure collection of sections containing the same neuroanatomical brain structures from all animals, according to a pig brain stereotaxic atlas (Félix et al., 1999). Endogenous peroxidase was quenched by incubation in 0.3% H_2_O_2_ (in PBS) for 10 min. Blocking of unspecific binding sites was performed with 1% BSA dissolved in PBS containing 0.05% Triton X-100. Sections were then incubated with primary antibodies (Supplementary Table S1) diluted in the blocking buffer. Primary antibody incubation (see Supplementary Table S1) was performed for 60 min at room temperature (RT, anti-doublecortin (DCX) labeling) or 16 h at 8°C (all other primary antibody incubations). The sections were subsequently incubated with species-specific horseradish peroxidase-conjugated secondary antibodies against mouse, rabbit, or goat IgG (Supplementary Table S1), for 30 min at RT. All antibody incubations were performed in moisture chamber. Antibody binding sites of the secondary HRP conjugated antibodies were visualized with a diaminobenzidine (DAB)-H_2_O_2_ reaction. Sections were then counterstained with Mayer’s hematoxylin. Samples from both groups were treated simultaneously to minimize inconsistency. The whole labeled sections were scanned at ×40 magnification (Hamamatsu, NanozoomerS60, Hamamatsu, Japan) and the specific regions of interest (ROIs) were delineated on each digital image (NDP.view 2 software, Hamamatsu Photonics, Hamamatsu, Japan) according to the pig brain stereotaxic atlas (Félix et al., 1999). Labeling consistency (background, intensity) was checked before quantification and labeling artefacts (e.g., tissue folds) were excluded from annotation. The ROIs were analyzed with ImageJ (NIH, Berthesda, MD, USA) and the fraction of immunoreactive area (area%) was digitally quantified for labeling of synaptophysin (SYN), myelin basic protein (MBP), DCX, allograft inflammatory factor 1 (Iba1), oligodendrocyte transcription factor 2 (Olig2), and neuronal nuclei (NeuN).

### Gene expression analysis

Frozen hippocampal, cerebellar, periventricular white matter, and caudate nucleus tissues were freeze-fractured in a cryogenic tissue pulverizer and homogenized in QIAzol lysis reagent (Qiagen, Copenhagen, Denmark). Total RNA was extracted using the RNeasy Lipid Tissue Mini kit (Qiagen, Copenhagen, Denmark) and 1 μg of RNA was reverse transcribed to cDNA using the high-capacity cDNA reverse transcription kit (Thermo Fisher, Waltham, MA, USA). Porcine-specific primers were designed with Primer-BLAST software (National Center for Biotechnology Information, USA) and were validated for efficiency and specificity (Supplementary Table S2). Relative gene expression was estimated by performing quantitative real-time PCR analysis on cDNA samples (10 ng) using LightCycler 480 SYBR Green I Master kit with porcine-specific primers on a LightCycler 480 machine (both Roche, Basel, Switzerland). Expression of target gene was normalized to the expression of housekeeping gene hypoxanthine phosphoribosyltransferase 1 (*HPRT1*) and presented as relative expression to the Control group as determined with the 2^−△△CT^ method.

### Statistics

Statistical analysis was performed using the R software (version 4.1.2, Vienna, Austria). Effect of IGF-1 treatment on protein synthesis rate, immunohistochemical, gene expression, beam test, gait performance, open field, and novel object recognition data were analyzed using a linear model with litter, birth weight, and sex as covariates. Effect of IGF-1 treatment on repeated measurement of home cage activity was analyzed using a linear mixed effects model with the same covariates as mentioned above in addition to the pig as a random effect, and an interaction term between time and treatment. Time for acquisition of operant conditioning (as defined by the learning criteria) was analyzed using a generalized linear mixed effects model, while time for acquisition of basic motor skills and survival probability were analyzed using a cox proportional hazard model, both statistical models included same cofactors as the linear model. Normal distribution and homoscedasticity of the residual and fitted values were tested for model validation, and data were log transformed when required. If log transformation of the data did not result in normal distribution and homoscedasticity of residuals a nonparametric analysis was used. Probability values <0.05 were considered statistically significant, while values between 0.1 and 0.05 were reported as a tendency toward an effect. Correlation analysis was performed using a Pearson R correlation. A one-sample Student *t*-test was used to compare the novel object reference index to a null preference value of 0.5.

Sensitivity analyses were conducted by removing animals in the lower 25th percentile for birth weight across groups to account for possible survival bias of smaller animals due to treatment. Additional sensitivity analyses were conducted by removing animals in the lower 25th percentile for growth rate (g/kg/day) across groups to account for possible survival bias due to treatment of *weaker* animals as defined by slow growth.

## Results

### Limited effects of IGF-1 supplementation on brain volume

**Figure 2:**
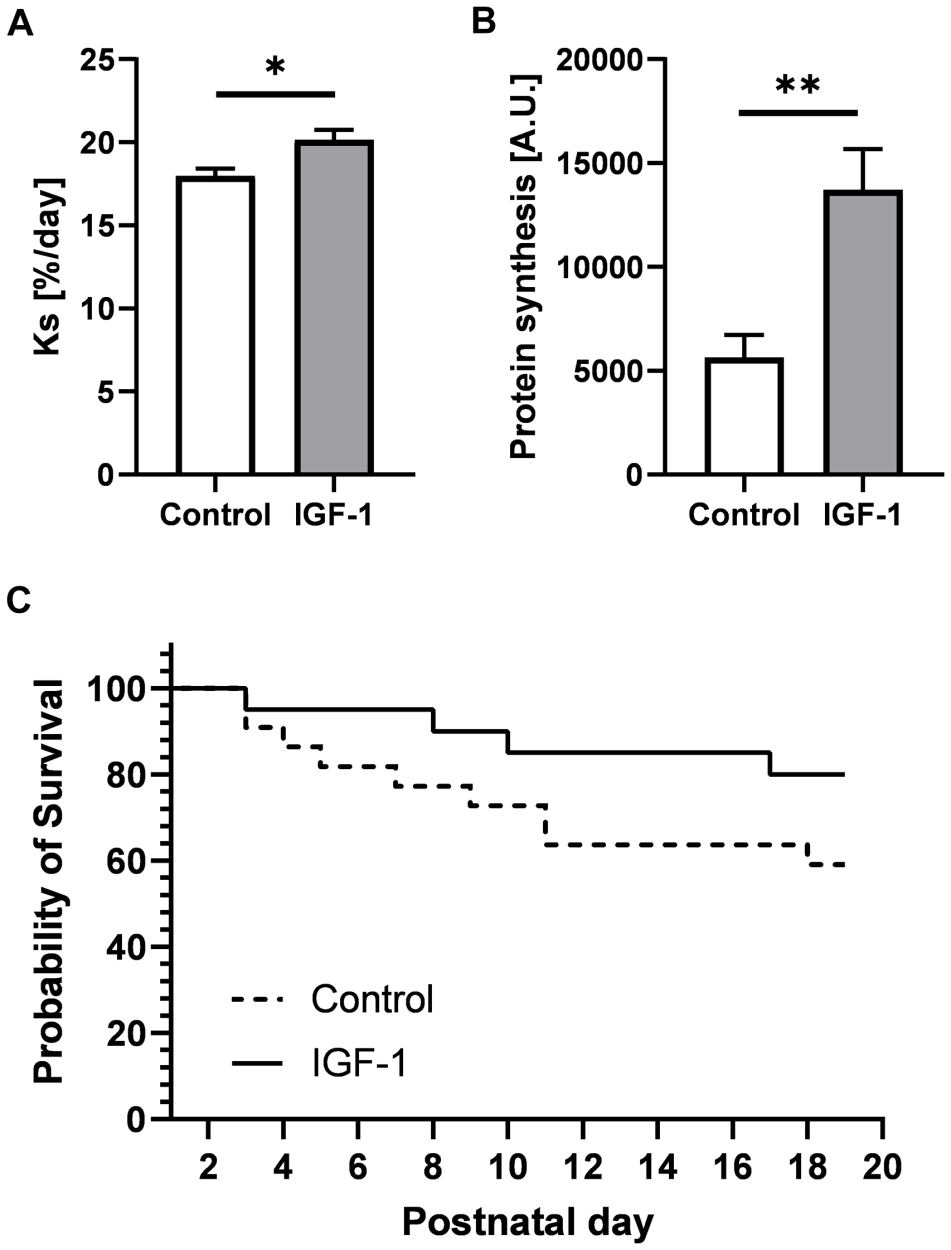
Effects of IGF-1 treatment on cerebellar protein synthesis rate and survival rate. **(A)** Protein synthesis rate measured by *in vivo* method for Control (*n* = 12, m/*f* = 8/4) and IGF-1 pigs (*n* = 14, m/f = 8/6). **(B)** Protein synthesis rate measured by *ex vivo* method for Control (*n* = 6, m/*f* = 4/2) and IGF-1 pigs (*n* = 10, m/*f* = 5/5). **(C)** Probability of survival. Control: group receiving vehicle, IGF-1: group receiving rhIGF-1/rhIGFBP3. Data is presented as means ± SEM for protein synthesis rate. *p < 0.05, **p < 0.001.

IGF-1 is an essential factor for postnatal growth and organ development, including gain in brain volume (Hellström et al., 2016, 2022). Total body and brain weights, including absolute and relative weights of specific brain regions, and water content was not affected by IGF-1 treatment (Table 1). However, absolute, and relative weights of the caudate nucleus region were reduced in IGF-1 pigs (*p* < 0.05 and *p* < 0.01, respectively). Male compared to female pigs had larger total cerebellum weight (3.60 ± 0.34, *n* = 18 vs. 3.35 ± 0.30, *n* = 12; *p* < 0.05) and relative cerebellum weight (10.16 ± 0.79, *n* = 18 vs. 9.82 ± 0.68; *n* = 12; *p* < 0.05), while their relative cerebrum weights were smaller than females (77.40 ± 0.20, *n* = 17 vs. 78.97 ± 0 0.39, *n* = 12; *p* < 0.01). Birth weight positively affected the final body weight, relative brain weight (*p* < 0.001, both), the total brain, and total cerebrum weights (*p* < 0.05, both). The rate of cerebellar protein synthesis measured *in vivo,* and *ex vivo* indicated brain anabolic effects of IGF-1 (*p* < 0.05 and *p* < 0.001, respectively, Figures 2A,B). Preterm pigs like preterm infants are predisposed to several morbidities due to organ immaturity, however, there was a tendency for IGF-1 treatment to increase the overall survival rate (64% for Control and 76% for IGF-1 pigs, *p* = 0.06; Figure 2C). Further, there was significant effect of birth weight on survival (*p* < 0.001), indicating a positive correlation between survival and birth weight especially for the Control group (Supplementary Figure S1).

**Table 1:** Total and relative weights of total brain and separate brain regions in control and IGF-1 pigs at PND19.

		CON	IGF-1	Value of *p*
Number of animals (m/f)		14 (8/6)	16 (10/6)	
Body weight	g	1924.21 ± 83.53	1809.75 ± 77.04	0.897^a^
Total brain	g	35.27 ± 0.58	35.49 ± 0.47	0.393^a^
Total brain	%	1.88 ± 0.08	2.02 ± 0.10	0.696^a^
Cerebrum	g	27.68 ± 0.50^b^	27.64 ± 0.41	0.544^a^
Cerebrum	%	78.27 ± 0.42^b^	77.87 ± 0.29	0.684^c^
Cerebellum	g	3.52 ± 0.11	3.48 ± 0.07	0.900^d^
Cerebellum	%	9.97 ± 0.25	9.82 ± 0.17	0.573^e^
Hippocampus	g	0.62 ± 0.02	0.60 ± 0.02	0.528
Hippocampus	%	1.75 ± 0.04	1.68 ± 0.04	0.204
Brain stem	g	3.52 ± 0.08	3.70 ± 0.05^f^	0.058
Brain stem	%	9.99 ± 0.20	10.46 ± 0.15^f^	0.120
Caudate nucleus	g	0.34 ± 0.01	0.31 ± 0.01	0.013*
Caudate nucleus	%	0.98 ± 0.03	0.88 ± 0.03	0.003**
Water content	%	82.82 ± 0.63	82.93 ± 0.95	0.853

The relative weight (%) of total brain is relative to total body weight, the relative weight (%) of brain regions are relative to total brain weight. Body weight was weighed on PND19 before euthanization. ^a^Effect of birth weight: Body weight (*p* < 0.001); Total brain, g (*p* = 0.029); Total brain, % (*p* < 0.001); Cerebrum, g (*p* = 0.034). ^b^*n* = 13 (m/f = 7/6). ^c^Effect of sex: *p* = 0.002; Female (78.97 ± 0.39, *n* = 12); Male (77.40 ± 0.20, *n* = 17). ^d^*p* = 0.042, Female (3.35 ± 0.30, *n* = 12), Male (3.60 ± 0.34, *n* = 18). ^e^Effect of sex: *p* = 0.016; Female (9.82 ± 0.68, *n* = 12); Male (10.16 ± 0.79, *n* = 18). ^f^*n* = 15 (m/f = 9/6). No other effects of covariates were observed. Data is presented as means ± SEM. *p < 0.05, **p < 0.001.

### IGF-1 supplementation affects locomotor development by improving balance and coordination

**Figure 3:**
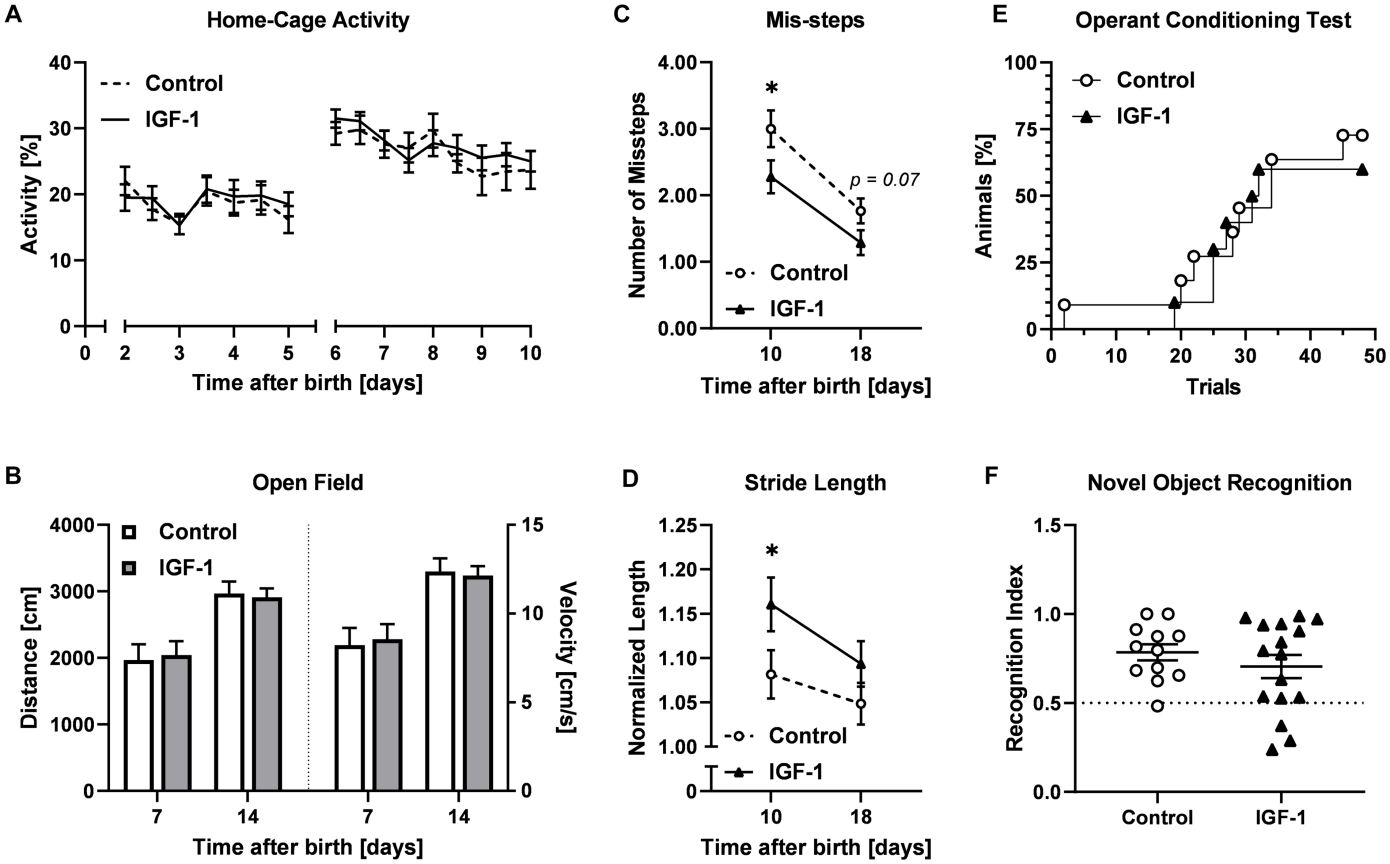
Effects of IGF-1 treatment on development of motor and cognitive functions. **(A)** Home cage activity for Control (*n* = 20, m/*f* = 10/10) and IGF-1 group (*n* = 19, m/f = 10/9) visualized as the proportion of active time. **(B)** Distance traveled and average velocity measured in the Open field test on PND7 and PND14 for Control (PND7: *n* = 18, m/*f* = 9/9; PND14: *n* = 13, m/*f* = 7/6) and IGF-1 pigs (PND7: *n* = 15, m/f = 8/7; PND14: *n* = 16, m/f = 9/7). **(C)** Number of missteps and **(D)** normalized stride length as quantified in the balance beam test on PND10 and PND18 for Control (PND10: *n* = 14, m/*f* = 6/8; PND18: *n* = 12, m/f = 7/5) and IGF-1 pigs (PND10: *n* = 15, m/f = 7/8; PND18: *n* = 15, m/f = 9/6). **(E)** Percentage of animals reaching the learning criteria in the operant conditioning test for Control (*n* = 11, m/f = 7/4) and IGF-1 pigs (*n* = 12, m/f = 6/6). **(F)** Recognition index of Control (*n* = 12, m/f = 7/5) and IGF-1 pigs (*n* = 16, m/f = 9/7) in the novel object recognition test. Control: group receiving vehicle, IGF-1: group receiving rhIGF-1/rhIGFBP3. Data is presented as means ± SEM. *p < 0.05.

Preterm pigs, unlike term-born, are not immediately able to walk and show general delayed motor development (Holme Nielsen et al., 2019). Acquisition of essential neuromotor control such as ability to open eyes, stand, walk, and drink from a feeding trough were not different between the Control and IGF-1 group (Supplementary Figure S2). Pigs in both groups were able to open their eyes, stand and walk within five days (Supplementary Figure S2A–C), and the pigs could drink from a trough within three days after being presented to it (Supplementary Figure S2D). The activity of the pigs in their home cage, measured as the relative time active in 12 h intervals, was comparable between the groups (Figure 3A). For both groups the relative activity increased on PND5 when the pigs were moved from incubators into larger home cages. At PND7 and PND14, pigs were tested to assess the development of basic locomotor function over time using an open field test (Figure 1). Pigs from both groups traveled similar distances and had similar average velocity in the arena at the two timepoints (Figure 3B).

The development of fine locomotor skills including balance and coordination was assessed in the balance beam test on PND10 and PND18 (Figure 3C). Pigs in the IGF-1 group had fewer average mis-steps compared to the Control at both time points (PND10, *p* < 0.05; PND18, *p* = 0.07). Analysis of the gait parameters showed that the IGF-1 group took significantly longer steps on the beam at PND10, however the effect was less pronounced at PND18 (PND10, *p* < 0.05; PND18, *p* > 0.05, Figure 3D).

### Cognition and memory were not affected by IGF-1 supplementation

Associative learning was analyzed using the operant conditioning test on PND12-19. The percentage of pigs reaching the learning criteria over time was similar between groups (Figure 3E). There was a significant effect of sex and birth weight with males reaching the learning criteria faster than females and pigs were faster with increasing birth weight (*p* < 0.001, both).

In the novel object recognition test, the capability of non-spatial short-term memory of the pigs was evaluated. For both groups the recognition index was significantly above the null preference value of 0.5 (Control: *p* < 0.001; IGF-1: *p* < 0.05), indicating a preference for the new object, but no differences were observed between the groups (Figure 3F). There was a significant effect of birth weight, indicating increasing recognition index value with increasing birth weight and suggesting reduced preference for the new object among low birth weight animals (*p* < 0.05).

### Decreased myelin basic protein expression without effects to gross white matter composition

**Figure 4:**
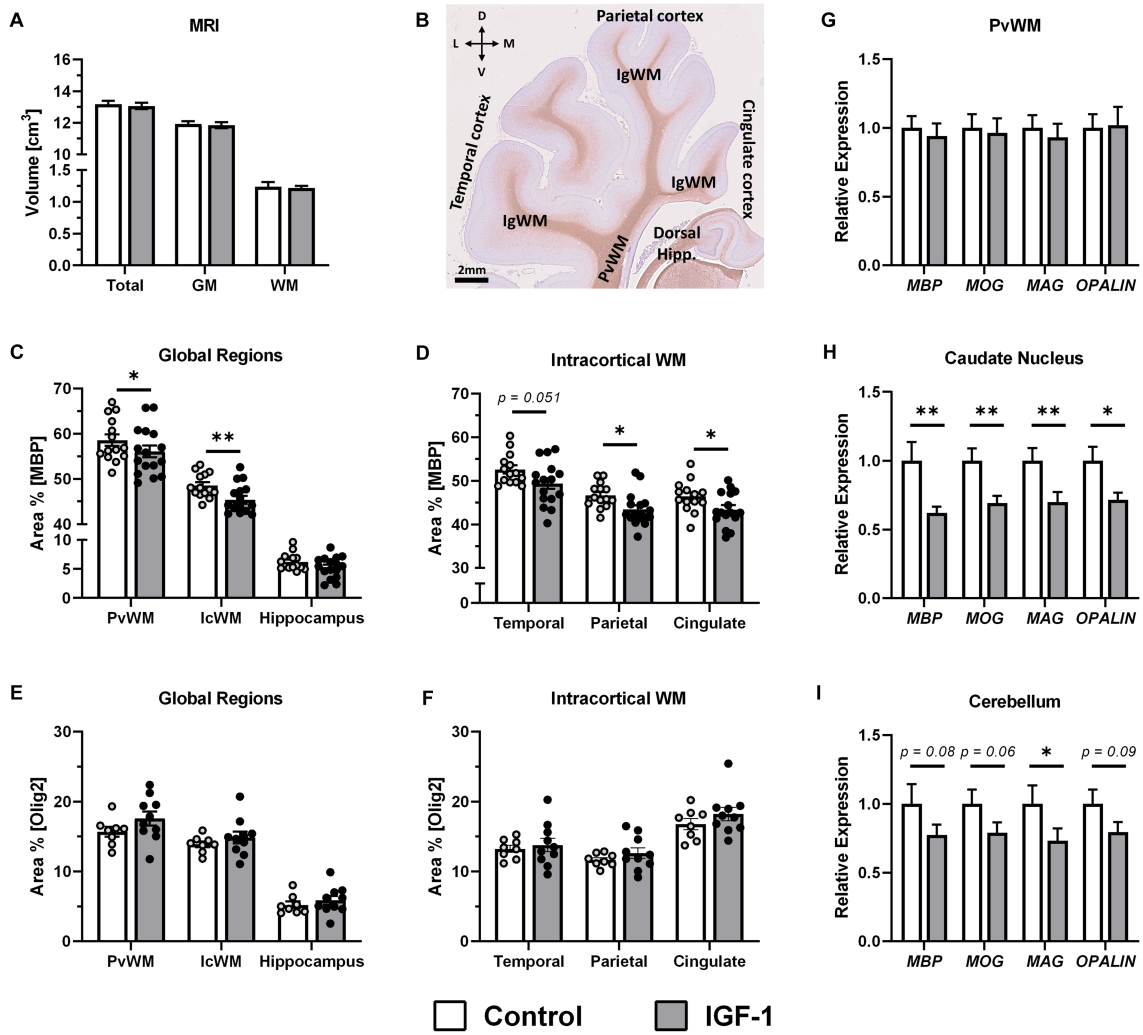
Effect of IGF-1 treatment on myelination. **(A)** Total brain, grey matter (GM) and white matter (WM) volume for Control (*n* = 14, m/f = 8/6) and IGF-1 pigs (*n* = 16, m/f = 10/6) as measured by magnetic resonance imaging (MRI). **(B)** Representative image of MBP/hematoxylin-labeled section illustrating selected regions of interest analyzed: intracortical white mater (IcWM), temporal cortex, parietal cortex, cingulate cortex, periventricular white matter (PvWM), and hippocampus (Hipp.). L: lateral, D: dorsal, M: medial, V: ventral. Quantification of the intensity of immunoreactivity as the proportion of area in the region of interest for MBP labeling in **(C)** global regions and **(D)** subregions of IcWM; Control (*n* = 14, m/f = 8/6) and IGF-1 (*n* = 16, m/f = 10/6) and for Olig2 labeling in **(E)** global regions and **(F)** subregions of IcWM; Control (*n* = 8, m/f = 5/3), IGF-1 (*n* = 10, m/f = 5/5). Expression of myelin associated genes in IGF-1 pigs (*n* = 12–16, m/f = 7–10/5–6) relative to Control pigs (*n* = 12–14, m/f = 7–8/5–6) as analyzed by qPCR in **(G)** PvWM, **(H)** caudate nucleus, and **(I)** cerebellum. Control: group receiving vehicle, IGF-1: group receiving rhIGF-1/rhIGFBP3. Data is presented as means ± SEM. *p < 0.05, **p < 0.001.

Grey and white matter volumes, measured by MRI, did not differ between groups (Figure 4A). To investigate if treatment had an effect on myelination in selected brain regions, the area of immunoreactivity (ir) of MBP, a marker of myelination and oligodendrocyte differentiation, was quantified in three regions of interest (ROIs): PvWM, intracortical white matter (IcWM), and hippocampus (Figure 4B). Comparison of MBP-ir in these ROIs showed significantly lower levels in PvWM and IcWM (*p* < 0.05 and *p* < 0.01, respectively) for the IGF-1 group, and similar levels in the hippocampus (Figure 4C). There was a significant effect of sex on MBP-ir in the hippocampus where males had higher levels compared to females (6.26 ± 0.37 vs. 4.88 ± 0.45, *p* < 0.05). Subdivision of IcWM into cingulate, parietal, and temporal subcortical areas showed that both the parietal and cingulate ROIs had significantly lower levels of MBP-ir in the IGF-1 group (*p* < 0.05, both), while the temporal ROI showed a tendency to a decreased level (*p* = 0.051, Figure 4D). Further analysis of the oligodendrocyte population was conducted by quantification of Olig2-ir, a pan-oligodendrocyte marker, in the same ROIs as for MBP-ir. Compared with controls, IGF-1 pigs had similar levels of Olig2-ir for all specified ROIs (Figures 4E,F).

Gene expression analyses of myelin protein related genes: *MBP*, myelin oligodendrocyte glycoprotein (*MOG*), myelin-associated glycoprotein (*MAG*); oligodendrocytic myelin paranodal and inner loop protein (*OPALIN*), showed no differences between groups in PvWM (Figure 4G). However, decreased expression of these genes was measured in the caudate nucleus for *MBP* (*p* < 0.01), *MOG* (*p* < 0.01), *MAG* (*p* < 0.01) and *OPALIN* (*p* < 0.05, Figure 4H). In cerebellum, expression of these genes was also lower for the IGF-1 group compared to Control, significantly for *MAG* (*p* < 0.05) although only with tendencies to significance for *MBP* (*p* = 0.08), *MOG* (*p* = 0.06) and *OPALIN* (*p* = 0.09; Figure 4I). There were no differences between groups in expression of the selected genes in the hippocampus (Supplementary Table S3).

### Neuronal maturation is promoted by IGF-1 treatment in caudate nucleus but unaffected in hippocampus

**Figure 5:**
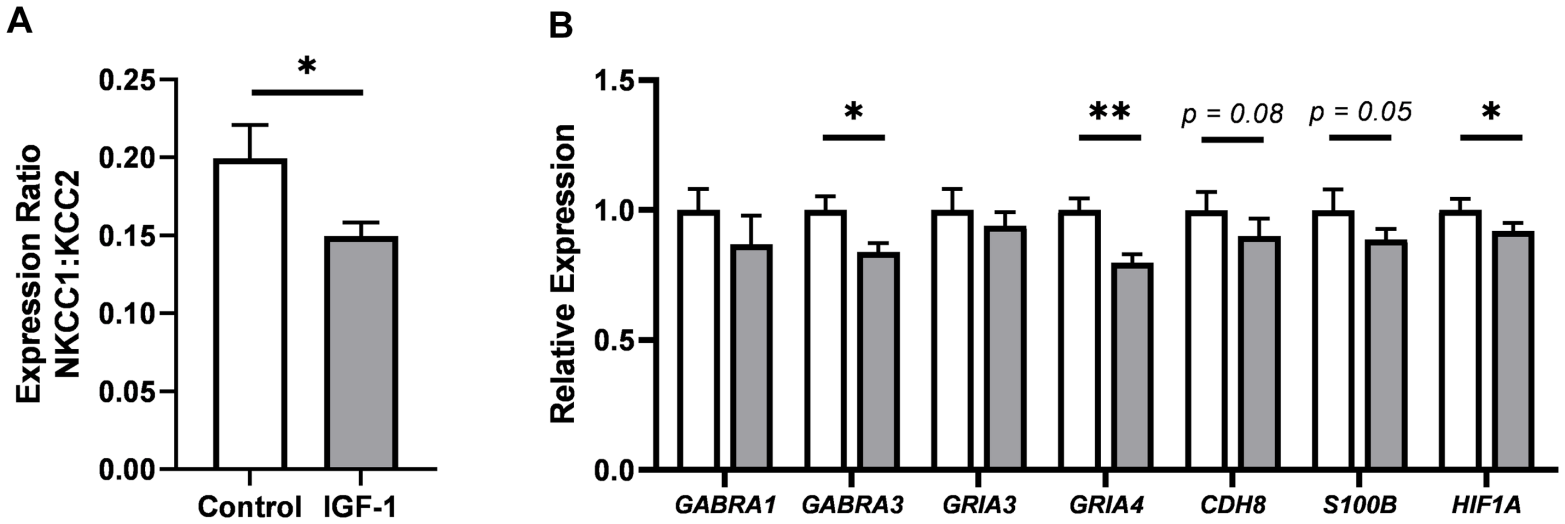
Effects of IGF-1 treatment on neuronal maturation in the caudate nucleus as measured by qPCR. **(A)** Gene expression ratio of NCKK1 over KCC2. **(B)** Expression of selected genes associated with neuronal maturation, illustrated as expression in IGF-1 pigs relative to Control. Control: group receiving vehicle (*n* = 12–13, m/f = 7–8/5), IGF-1: group receiving rhIGF-1/rhIGFBP3 (*n* = 15–16, m/f = 9–10/5–6). Data is presented as means ± SEM. *p < 0.05, **p < 0.001.

Based on the specific effect of the treatment on caudate weight and expression of myelination-related genes, the effect on neuronal maturation in the caudate nucleus was also investigated. The expression of a panel of genes was quantified to reflect different aspects of neuronal system development (Supplementary Table S3). Interestingly, the ratio of sodium-potassium-chloride cotransporter 1 (*NKCC1*) over potassium-chloride cotransporter 2 (*KCC2*) expression, an indicator of maturation of the gamma-aminobutyric acid (GABA) signaling system, was significantly decreased for IGF-1 vs. Control pigs (Figure 5A, *p* < 0.05), suggesting progressed maturation of the GABAergic system. A significant decrease was also seen in the gene expression of GABA receptor subunit alpha 3 (*GABRA3*) for the IGF-1 group (Figure 5B, *p* < 0.05), further supporting an IGF-1 treatment effect on the GABAergic system. Decreased IGF-1 related expression was also seen for glutamate receptor 4 (*GRIA4*, *p* < 0.01) and hypoxia-inducible factor 1-alpha (*HIF1A*, *p* < 0.05), with tendencies for cadherin 8 (*CDH8*, *p* = 0.08) and S100 calcium-binding protein B (*S100B*, *p* = 0.05). The expression of genes related to IGF-1 signaling were similar between groups (Supplementary Table S3). Overall, the gene expression analyses indicated IGF-1 effects on neuronal maturation, especially with regards to the GABAergic system, but without effects on endogenous IGF-1 signaling in the caudate nucleus.

**Figure 6:**
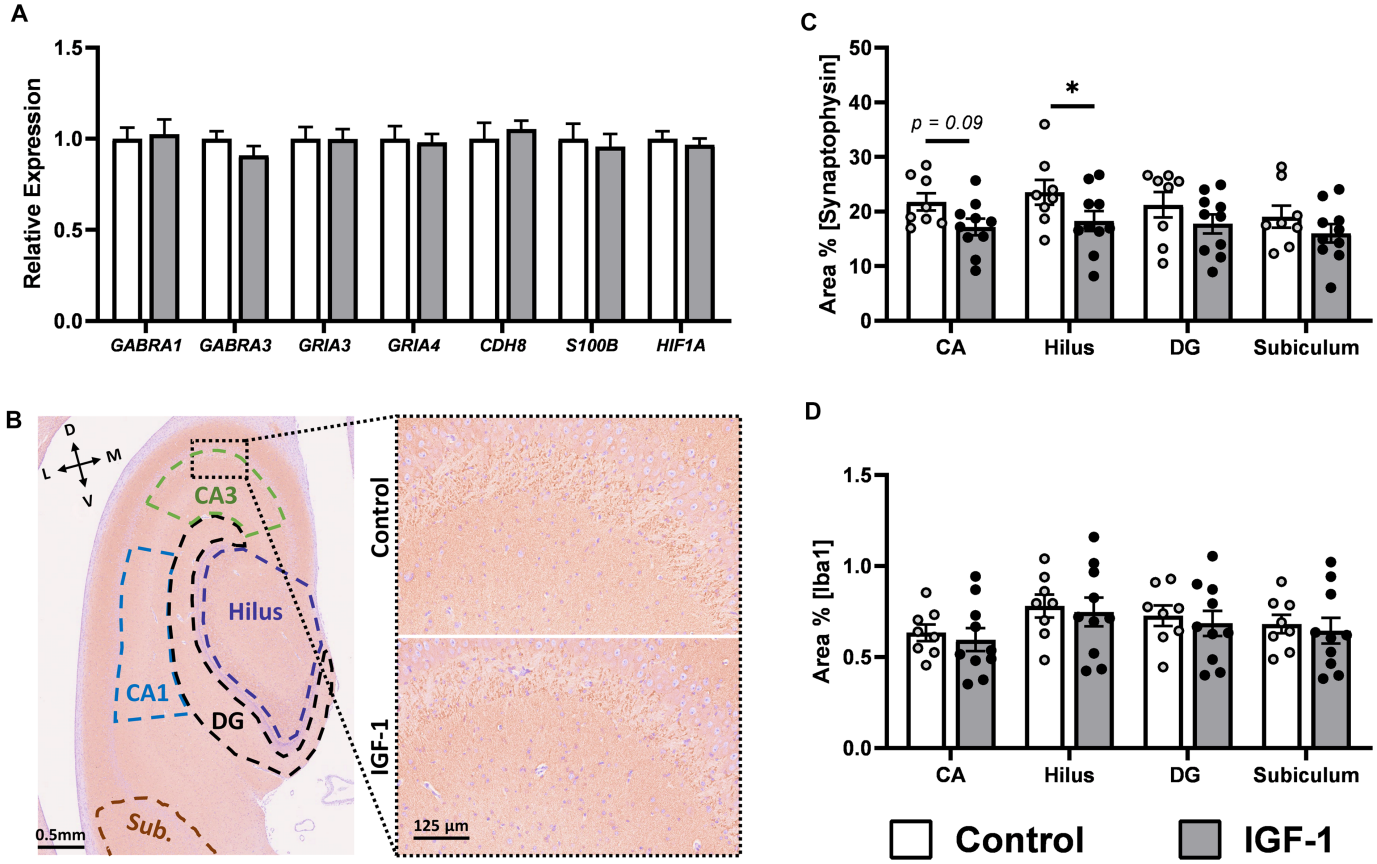
Neuronal maturation in hippocampus. **(A)** Expression of selected genes associated with neuronal maturation in IGF-1 pigs (*n* = 14–16, m/f = 8–10/5–6) relative to Control pigs (*n* = 11–13, m/f = 6–8/4–5) as analyzed by qPCR. **(B)** Representative image of SYN/hematoxylin-labeled section illustrating selected regions of interest analyzed: subiculum (Sub; brown), *cornu ammonis* 1 (CA1; blue), CA3 (green), hilus (purple), dentate gyrus (DG; black). Inserts are enlarged representative images of pyramidal cell and stratum lucidum layer in CA3. L: lateral, D: dorsal, M: medial, V: ventral. Quantification of the intensity of immunoreactivity as the proportion of area in the region of interest for **(C)** synaptophysin and **(D)** Iba1 labeling in hippocampal subregions for Control (*n* = 8, m/f = 5/3) and IGF-1 pigs (*n* = 10, m/f = 5/5). Control: group receiving vehicle, IGF-1: group receiving rhIGF-1/rhIGFBP3. Data is presented as means ± SEM. * *p* < 0.05, ** *p* < 0.001.

For hippocampus, previous studies show that the IGF-1 receptor is highly expressed in this region and co-localized with immature neurons in preterm pigs (Christiansen et al., 2023). However, the expression level of a panel of genes related to general neuronal maturation and development showed no differences between groups (Figure 6A; Supplementary Table S3). There were no effects on the hippocampal neuron population, as examined by NeuN-ir, a marker of mature neurons, and DCX-ir, a marker of immature neurons (Supplementary Figure S3). In contrast, quantification in hippocampal subregions (Figure 6B) of SYN-ir, a membrane protein of synaptic vesicles and marker of synaptic density, showed lower levels in the hilus subregion of the hippocampus for the IGF-1 group (Figure 6C, *p* < 0.05) and a tendency for this effect in the CA subregion (Figure 6C, *p* = 0.09). Iba1-ir (a marker of microglia) was similar between groups, measured for the same regions as SYN-ir, indicating no microgliosis here (Figure 6D).

### Sensitivity analyses adjusting for birth weight and postnatal growth rates demonstrates robustness of treatment effects

The tendency to higher survival for IGF-1 pigs, with significant effect of birth weight, suggested a survival bias for smaller animals in the IGF-1 group. Sensitivity analyses were therefore conducted by removing the lowest 25th percentile for birth weight for both treatment groups (Supplementary Table S4), accepting lower, but more homogenous, sample size for comparison. Most findings were unchanged after this adjustment, however the previous significant effects (full sample size) of IGF-1 on MBP-ir (PvWM, cingulate subcortex), stride length (beam test) some caudal gene expressions (MOG, MAG, OPALIN, NKCC1/KCC2 ratio) disappeared after exclusion of the low birth weight pigs in the sensitivity analysis, although group means ± SEM remain similar to the primary analyses.

The increased risk of mortality for pigs with lower birth weight reflects their increased risk of severe clinical complications (Supplementary Figure S1). Animals that suffer from these complications show poor growth and sensitivity analyses were therefore also conducted by removing the lower 25th percentile for growth rate. Again, treatment effects remained mostly unchanged but after exclusion of pigs with lowest growth rate, the differences in levels of MBP-ir (PvWM, parietal and cingulate subcortex) between groups were no longer significant. Further, IGF-1 treatment failed to affect stride length (beam test) and reduced the percentage of animals reaching the learning criteria in the operant conditioning test. Although, these results were based on a lower sample size.

## Discussion

Understanding the effects of IGF-1 treatment on early brain development is important to evaluate safety and efficacy for IGF-1 supplementation after preterm birth, currently being investigated in extremely preterm infants (Clinicaltrials.gov registry NCT03253263). In our previous studies, IGF-1 treatment stimulated brain protein synthesis and maturation of both grey and white matter during the first 5–9 d after preterm birth (Christiansen et al., 2023). This is a period of high clinical sensitivity of preterm pigs characterized by developmental immaturities such as cardiovascular and metabolic instability, gut and immune dysfunctions, and poor neuromuscular control (Sangild et al., 2013; Andersen et al., 2016; Holme Nielsen et al., 2019; Bæk et al., 2021). In this study, we extended IGF-1 supplementation to three weeks of life, an age when preterm pigs start to function and grow normally, following their clinical challenges in the first weeks, thus allowing a series of neurofunctional tests. We show that IGF-1 treatment improved fine motor balance and coordination, together with indications of enhanced maturation of the GABAergic system in the caudate nucleus. To our surprise, long-term IGF-1 treatment decreased caudate nucleus weight and expression of myelination-related genes in this region (with a tendency also in the cerebellum), and the level of MBP-ir was reduced in white matter regions but without effects to total white and grey matter volumes. The differential effects of IGF-1 supplementation observed in this study and our previous studies show that the effects of postnatal IGF-1 supplementation on the immature preterm brain may be highly dependent on the treatment initiation time and period, potentially interacting with clinical status and degree of immaturity. We suggest that supplemental IGF-1 may be most important in the first 1–2 weeks after preterm birth, at least in pigs. At this time, IGF-1 treatment may improve survival especially of the smallest/weakest pigs and when we excluded such pigs in our post-hoc sensitivity analyses, some of the brain effects disappeared. Correspondingly, effects of IGF-1 supplementation to preterm infants may depend on the treatment time, length, gestational age but also weight at birth and clinical complications.

Preterm infants are highly susceptible to white matter injury which can lead to neurodevelopmental delay such as motor dysfunctions due to differential arrest of oligodendrocytes and myelination failure (Back et al., 2002; Back, 2017). Following three weeks of IGF-1 treatment, the level of MBP-ir was lower in both PvWM and IcWM compared to non-treated animals. In contrast, qPCR analysis of genes associated with myelination (*MBP, MOG, MAG, OPALIN*) showed no effect of IGF-1 treatment in PvWM, indicating that myelin synthesis may be affected at protein level. IGF-1 treatment did not affect Olig2-ir in white matter ROIs, suggesting no effect of the treatment to the total number of oligodendrocytes (Olig2 is a pan-oligodendrocyte marker). It can therefore not be ruled out that decreased myelin synthesis is a result of decreased maturation of oligodendrocytes to mature myelin producing cells. However, this is in contrast with our previous study and several rodent-based studies where exogenous IGF-1 advanced oligodendrocyte differentiation, proliferation, survival and myelin development (Beck et al., 1995; Ye et al., 1995, 2002; Gao et al., 1999; Lin et al., 2005; Zeger et al., 2007; Cai et al., 2011). Direct comparison of brain development between species remains difficult but the perinatal brain growth spurt, regional brain anatomy and grey-to-white matter ratio appears more similar between infants and pigs, relative to young rodents (Dobbing and Sands, 1979). In pigs, myelin development in the perinatal period has two peaks, the first about two weeks before birth and the second about three weeks after birth (Sweasey et al., 1976). Such biphasic myelination process may explain the differential effects of supplemental IGF-1 on myelination at different postnatal ages (e.g., increased MBP initially and decreased levels later). In addition, it cannot be excluded that the longer treatment period in this study induced a desensitization of brain IGF-1 receptors, in line with reports of opposite effects from high-and low-dose IGF-1 (Cao et al., 2003; Pang et al., 2010). Although, it has been shown that IGF-1 can cross the blood brain barrier (BBB) (Reinhardt and Bondy, 1994; Pan and Kastin, 2000; Nishijima et al., 2010), alterations in the expression of transporters facilitating the passage of this peptide across the BBB and thus availability of supplemental IGF-1 in the brain during maturation may also explain the differences in myelination between short and longer term treatment (Lee et al., 1989; Moretti et al., 2015). However, the decrease in MBP level in white matter regions (primary analyses) did not affect the gross volume of white matter or total brain weight as expected if global myelination was affected.

Our longer-term treatment of pigs with IGF-1 specifically affected the absolute and relative weight of the caudate nucleus, without affecting the total brain weight. IGF-1 treatment also decreased the expression of myelin associated genes in caudate tissue, while there was a tendency for the same effect in the cerebellum but no effect in the hippocampus, suggesting that the caudate nucleus was particularly sensitive to the IGF-1 treatment, or it may reflect the bi-phasic striatal expression of myelination genes during early postnatal development reported in rats (Novak et al., 2013). In humans, the caudate nucleus is associated with planning and execution of movement, but also with several other higher neurological functions such as cognition and emotion (Grahn et al., 2008). In children, caudate volume development and later IQ does not show any consistent relation (Giedd et al., 1996; Abernethy et al., 2004; Raznahan et al., 2014; Wierenga et al., 2014). The development of caudate volume has not previously been described in pigs but a study of Yucatan minipigs confirmed the critical role of the caudate nucleus in locomotion (Coquery et al., 2019). We detected no IGF-1 effects on basic motor skills or locomotion in open field tests. However, gait parameters and subsequent performance were improved in the IGF-1 group during the balance beam test, suggesting an association between caudate function and more advanced motoric development in relation to movement and coordination. The pig can be considered a precocial species (e.g., fast maturation shortly after birth) with regards to locomotion and specifically motor functions may be a principal phenotypic target for factors stimulating neurological development. On the other hand, changes in caudate volume may be secondary to IGF-1 effects on other brain regions or systems that might be functionally or structurally changed by the treatment.

To further investigate changes in caudate function, gene expression analysis was performed for caudate tissue. Interestingly, our data showed that IGF-1 administration promoted a decrease in gene expression of *GABRA3*, *GRIA4*, and *NKCC1:KCC2* ratio in the caudate tissue, suggesting that treatment influenced the GABAergic system. According to a previous study, the expression of *GABRA3* in the pig basal ganglia peaks at embryonic day 100 and is developmentally downregulated at PND4 (Miller et al., 2017), which is approximately equivalent to preterm PND19. The slight but significant decrease of *GABRA3* expression in the caudate nucleus of IGF-1 pigs further indicates the maturation effect of IGF-1 supplementation. GABA is the main inhibitory neurotransmitter in the central nervous system, however it is well known that in the developing nervous system GABA instead has an excitatory effect which is essential for proliferation, migration, differentiation, and plasticity of developing neurons (Ben-Ari et al., 2007; Peerboom and Wierenga, 2021). The developmental shift in GABA function is mediated by a change in the expression of chloride (Cl^−^) transporters NKCC1 (importer) and KCC2 (exporter), with a high NKCC1:KCC2 ratio in immature neurons and low ratio in mature neurons (Ben-Ari et al., 2007; Peerboom and Wierenga, 2021). Our data showed that our longer-term treatment of preterm pigs with IGF-1 decreased the ratio of NKCC1:KCC2, which is mainly driven by decreasing the *NKCC1* expression, thereby possibly promoting the postnatal GABA shift toward the inhibitory function of mature neurons, in accordance with previous rodent and *in vitro* studies (Kelsch et al., 2001; Baroncelli et al., 2017). The accelerated GABA shift in IGF-1 treated pigs coincided with improved development of balance and motor coordination, thus supporting a study in mice showing an association between maturation of striatal medium spiny neurons and onset of quadruped motor development (Dehorter et al., 2011). Furthermore, IGF-1 might improve the maturation of the visual system *via* an accelerated postnatal shift in GABAergic signaling (Baroncelli et al., 2017)^,^ thus improved performance of the pigs in the balance beam test might be explained by advanced visual maturation. This notion is supported by imaging studies of human adult brains showing that the caudate nucleus interacts with the inferior temporal lobe to process visual information and coordinate movement accordingly (Seger and Cincotta, 2005; Graff-Radford et al., 2017). Future studies should address how accelerated GABA system maturation, myelination, and caudate neuron development interact to influence movement and coordination in preterm infants with and without IGF-1 supplementation.

The hippocampus and cerebellum are both brain regions which communicate with the caudate nucleus and these networks are important for cognitive and motor functions, respectively (Grahn et al., 2008). Our data showed no effect of IGF-1 on neuronal maturation in these tissues, except a tendency to decrease expression of myelin associated genes in the cerebellum. Short-term IGF-1 treatment increased the level of hippocampal NeuN-ir in our previous study (Christiansen et al., 2023), yet in the current longer-term study there was no effect of IGF-1 on mature or immature neuron development and only minor effects on SYN-ir in hippocampal subregions. In accord, there was also no effect on cognitive performance, as estimated by the operant conditioning and novel object recognition test.

Preterm infants are vulnerable to neurodevelopmental insults that may disturb their normal trajectory of brain maturation, behavior, and locomotion. Our results show that supplementation with IGF-1 during the first three weeks after preterm birth in pigs promotes maturation of the GABAergic system in the caudate nucleus, a region associated with planning and execution of movement, supporting more advanced motor skills in these pigs in the beam test, without effects to total white matter volume, brain weight or performance in other neurofunctional tests. More studies are needed to determine the mechanistic effect of IGF-1 in the caudate region and how treatment for just 1–2 weeks after preterm birth affects later cognition and motor function.

Our study has several limitations, including the necessary concern related to direct comparison between pigs to infants with regards to brain ontogeny and structural/functional neurological development. Despite these uncertainties, our results provide some basic information combining a clinically-and physiologically relevant supplementation of IGF-1 with all known complications of preterm birth (immature respiratory, digestive, metabolic, immune and brain functions) in the same animal model. Survival bias was considered in our study because fewer pigs from the IGF-1 group were euthanized due to clinical complications, thus leaving more of the weaker (and often smaller) preterm pigs alive for tissue collection and behavioral testing in the IGF-1 group compared with Control pigs. This may affect the results investigating white matter development as preterm birth, clinical complications, and low birth weight increase the risk of white matter injury (Back, 2017). In line with these considerations, the effects of IGF-1 on periventricular and subcortical MBP levels were less pronounced or insignificant following sensitivity analyses possibly confirming that the IGF-1 effects to MBP levels are biased due to higher survival in this group. Although, the sensitive analyses are limited by small sample size which likely affects the significance of the results.

Collectively, our data supports the idea that supplementary IGF-1 in the postnatal period of preterm neonates enhances maturation of a range of body systems, including the central nervous system, and thereby critical capacities, such as motor function. More studies in both pigs and infants are required to define the optimal time, length and dose of treatment for the various groups of (extremely, very, moderate) preterm infants suffering various clinical complications.

## Data availability statement

The original contributions presented in the study are included in the article/Supplementary materials, further inquiries can be directed to the corresponding author.

## Ethics statement

The animal study was reviewed and approved by the Danish National Committee on Animal Experimentation (license no.: 2020-15-0201-00520) and complied with ARRIVE guidelines.

## Author contributions

PS, TT, and SP: conceptualization. PS: funding acquisition and project administration. LC, KA-O, JB-MS, TT, and SP: animal experiments. BH, GV, LC, SL, DB, YM, and SP: tissue analyses. ML: video analysis. LC: data analysis. LC, PS, and SP: writing and editing. All authors read and approved the final version.

## Funding

The University of Copenhagen received funding from Takeda Pharmaceutical Company, MA, USA and Oak Hill Bio. These funders were not involved in the study execution, collection, analysis, interpretation of data, the drafting of this article or the decision to submit it for publication.

## Conflict of interest

BH was employed by ImaGene-iT AB. The University of Copenhagen represented by authors TT, PS, and SP, and the company Oak Hill Bio have filed a patent application directed to use of rhIGF-1 for preterm infants.

The remaining authors declare that the research was conducted in the absence of any commercial or financial relationships that could be construed as a potential conflict of interest.

## Publisher’s note

All claims expressed in this article are solely those of the authors and do not necessarily represent those of their affiliated organizations, or those of the publisher, the editors and the reviewers. Any product that may be evaluated in this article, or claim that may be made by its manufacturer, is not guaranteed or endorsed by the publisher.

## Glossary

BBB: blood brain barrier
CDH8: cadherin 8
DCX: doublecortin
GABA: gamma aminobutyric acid
GABRA3: gamma aminobutyric acid receptor subunit alpha 3
GRIA4: glutamate receptor 4
HIF1A: hypoxia-inducible factor 1-alpha
HPRT1: hypoxanthine phosphoribosyltransferase 1
Iba1: allograft inflammatory factor 1
IcWM: intracortical white matter
IGF-1: insulin-like growth factor 1
IGF-1R: insulin-like growth factor 1 receptor
IGFBP3: insulin-like growth factor binding protein-3
IHC: immunohistochemistry
KCC2: potassium-chloride cotransporter 2
MBP: myelin basic protein
MAG: myelin-associated glycoprotein
MOG: myelin oligodendrocyte glycoprotein
MRI: magnetic resonance imaging
NeuN: neuronal nuclei
NKCC1: sodium-potassium-chloride cotransporter 1
Olig2: oligodendrocyte transcription factor 2
OPALIN: oligodendrocytic myelin paranodal and inner loop protein
PND: postnatal day
PvWM: periventricular white matter
rh: recombinant human
ROI: region of interest
S100B: S100 calcium-binding protein B
SYN: synaptophysin
ir: area of immuno reactivity
