# Supplementary material for: Insulin-like growth factor 1 supplementation supports motor coordination and affects myelination in preterm pigs
Source: Front Neurosci. 2023 Jun 19;17:1205819. doi: 10.3389/fnins.2023.1205819 (PMC10315495; doi:10.3389/fnins.2023.1205819)
Supplement: Supplementary file 1 [file Table_1.DOCX]

**Supplementary Table S1** List of antibodies, dilutions, and supplier information for immunohistochemistry.

| **Epitope** | **Source** | **Dilution** | **Supplier (#Catalog)** |
| --- | --- | --- | --- |
| *Primary antibody* | | | |
| DCX | Rabbit polyclonal | 0.4 μg/mL | Abcam (#ab18723) |
| IBA1 | Goat polyclonal | 1:750 | Abcam (#ab5076) |
| MBP | Mouse monoclonal | 1:50 | BioLegend (#836504) |
| NeuN | Mouse monoclonal | 1:150 | Merck (#MAB377) |
| Olig2 | Rabbit polyclonal | 1:200 | Merck (#AB9610) |
| Synaptophysin | Mouse monoclonal | 1:100 | Abcam (#ab8049) |
| *Secondary antibody* | | | |
| Rabbit IgG/HRP | Goat | 1:1 | Vector (#MP-7451) |
| Mouse IgG/HRP | Goat | 1:1 | Vector (#MP-7452) |
| Goat IgG/HRP | Donkey | 1:200 | Jackson IR (#705-036-147) |
