# Supplementary material for: Insulin-like growth factor 1 supplementation supports motor coordination and affects myelination in preterm pigs
Source: Front Neurosci. 2023 Jun 19;17:1205819. doi: 10.3389/fnins.2023.1205819 (PMC10315495; doi:10.3389/fnins.2023.1205819)
Supplement: Supplementary file 2 [file Table_2.DOCX]

**Supplementary Table S2.** List of primer sequences for qPCR.

| **Gene** | **Gene name** | **Primer sequences (5’ to 3’)** | **Product size (bp)** |
| --- | --- | --- | --- |
| *ALDOC* | Aldolase C | F: CCTTCGTCCGTACCATCCAG  R: GTTGTTTCTCCATCAGTCCCG | 94 |
| *AQP4* | Aquaporin 4 | F: GAGCCGGGATTCTCTACCTG  R: ATTTCCATGAACCGTGGTGA | 74 |
| *ATXN1* | Ataxin 1 | F: ACGCAACCCCAGATCCATTTA  R: ATGTGAGACACACTTCCCTGC | 169 |
| *CALB1* | Calbindin 1 | F: GGCCAGGTTACTACCAGTGC  R: TCCATTGCCATCCTGATCGT | 112 |
| *CALB2* | Calbindin 2 | F: GACGCAGACGGAAATGGGTA  R: ACACCATGCCAGAGCCTTTT | 91 |
| *CASP3* | Caspase 3 | F: GAGGGGACTGCTGTAGAACT  R: GTCCCACTGTCCGTCTCAAT | 103 |
| *CBLN1* | Cerebellin 1 precursor | F: ATCAGAAGCACCAACCACGA  R: GCGATGAAAGTGCTGCGTTC | 119 |
| *CDH8* | Cadherin 8 | F: GTTACATTTGGCAGGCAGGC  R: CAGCGGATGAGCTTCAAGGA | 100 |
| *CNMD* | Chondromodulin | F: CATGACGTTCGACCCTCGAC  R: CGGCAGCCTTGGTAGTTGTA | 135 |
| *DNM1* | Dynamin 1 | F: GAAGCGCATTGAAGGCTCAG  R: TGGCTTCAAAAGCGAGGTCA | 212 |
| *DRD1* | Dopamine receptor D1 | F: CCTCAAGGCAAGGGCTTT  R: TCACATTTACGGGCTGTTGC | 132 |
| *DRD2* | Dopamine receptor D2 | F: CAGAACGAGTGCATCATCGC  R: CGTGCAGTTGCCCTTGAGTG | 201 |
| *EEAF1A1* | Eukaryotic translation elongation factor 1 alpha 1 | F: TTGCATTCTACCACCAACTCGT  R: AACATTGACTGGAGCAAAGGTG | 157 |
| *EEAF1A2* | Eukaryotic translation elongation factor 1 alpha 2 | F: TCAGTCAAGGACATCCGTCG  R: CTTCTCCTTGAGCTCGGCAA | 189 |
| *EFNA5* | Ephrin A5 | F: TGTTTCTCGTGCTCTGGATGT  R: ACCCCTCTGGAATCTGGGGTT | 110 |
| *ERBB2* | Erythroblastic oncogene B 2 | F: TGCAGAGAACCCCGAATACC  R: CGATGTGGCCCAGACATCAG | 118 |
| *FGF2* | Basic fibroblast growth factor | F: TGGTACCTGCACCCCAATTT  R: TGTCCCCTTTCCCTACTGTT | 176 |
| *GABRA1* | Gamma-aminobutyric acid type A receptor subunit alpha 1 | F: TCTCTCGCAGACTTTTCCCG  R: AGGGTTGTCCATAGCTTCTTCC | 159 |
| *GABRA3* | Gamma-aminobutyric acid type A receptor subunit alpha 3 | F: GCCAAGGGGAATCAAGACGA  R: ATGCTTTGGAGAGAGTCCGC | 74 |
| *GAD1* | Glutamate decarboxylase 1 | F: CTACGCCGAAGAGCGACC  R: GAAGAAGGGGTCGAAGACGC | 322 |
| *GAD2* | Glutamate decarboxylase 2 | F: TGCAATCAAAACAGGGCATCC  R: TGCAGTTGATGTCAGCCAGT | 94 |
| *GAP43* | Growth associated protein 43 | F: GCCGAGGAGAAGATAGAAGCTGTA  R: TCCTTTACCCTCATCCTGCC | 72 |
| *SLC2A3* | Solute carrier family 2 member 3 (GLUT3) | F: TCCCCTCAGCTGCATTCTAT  R: CCAGAAGACAACGAGGAAGC | 71 |
| *GRIA3* | Glutamate receptor 3 | F: ACAGAACTTCGTTCTAGGCGT  R: AGAATGACCCAAAAGCCCCA | 102 |
| *GRIA4* | Glutamate receptor 4 | F: TCAGTGAGGCAGGCGTCTTA  R: AGCCGCCAACCAGAATGTAG | 150 |
| *HIF1A* | Hypoxia-inducible factor 1-alpha | F: TTTACTCATCCGTGCGACCA  R: AGCTCCGCTGTGTATTTTGC | 97 |
| *HPRT1* | Hypoxanthine-guanine phosphoribosyltransferase | F: ACACTGGCAAAACAATGCAA  R: TGCAACCTTGACCATCTTTG | 71 |
| *HTR2A* | 5-Hydroxytryptamine receptor 2A | F: TCCGTCAATGAAAAGCAGGGT  R: GCTCACAGCTGAAATGCTGTC | 77 |
| *IGF1*^¤^ | Insulin-like growth factor 1 | F: ATTTCTTGAAGGTAAAGATGCA  R: CAGCCCCACAGAGGGTCTCA | 117 |
| *IGF1R*^¤^ | Insulin-like growth factor 1 receptor | F: CGAGAGACATCTATGAGACA  R: TCCTCACTGTAGTAGAAGGA | 382 |
| *IGF2*^¤^ | Insulin-like growth factor 2 | F: CCCAGTGAGACTCTGTGCG  R: CAGGTGTCATAGCGGAAGAAC | 275 |
| *IGF2R*^¤^ | Insulin-like growth factor 2 receptor | F: ATCCTCAATCCCATAGCC  R: CTCTTACAATGAAACGCAAT | 110 |
| *IGFBP3*^¤^ | Insulin-like growth factor binding protein 3 | F: GACACGCTGAACCACCTCA  R: CGTACTTATCCACGCACCAG | 151 |
| *IRS* | Insulin receptor substrate 1 | F: TGGACATCACAGCAGAATGAAGA  R: GGTGTGAGGTCCTGGTTGTG | 105 |
| *MAG* | Myelin associated glycoprotein | F: CCGTGGGAAGAGAGTGTTGAA  R: AGTGAGCAACAGCTCCGTCT | 122 |
| *MBP* | Myelin basic protein | F: TGACTACAAACCGGCTCACA  R: TCCCAGCTTGAAGATTTTGG | 79 |
| *MLC1* | Megalencephalic leukoencephalopathy with subcortical cysts 1 | F: GCCTCCAGAGAAACGCAGTA  R: TTTCCCGTAGCCAAGAGACG | 115 |
| *MOG* | Myelin oligodendrocyte glycoprotein | F: AGCGCAGACTGAGAGGAAAAC  R: CCGAGAACTGGCACGATCA | 121 |
| *NEUROD1* | Neurogenic differentiation factor 1 | F:AGGACCTACTAACAACTACAGGAGA  R: GACACTCGTCTGTCCAGCTT | 111 |
| *NEUROD2* | Neurogenic differentiation factor 2 | F: GAGGGCTCCTTCGAGGTTTC  R: GTGTCCGACGGGAGTTTCAT | 191 |
| *NRP1* | Neuropilin 1 | F: CGGAACAGGTGATGACTTCCA  R: AGAACCCCAGCCAAACTCAC | 133 |
| *NTRK2* | Neurotrophic receptor kinase 2 (trkB) | F: TACGGTTTGTCACTCGACCC  R: ATGGAAACCCGTCTCGGTTC | 191 |
| *OPALIN* | Oligodendrocytic myelin paranodal and inner loop protein | F: CTGCTGGTGGCCTTACTGTT  R: TTCACAAGGCCTCTCGATTT | 84 |
| *PCLO* | Protein piccolo | F: AGTTCCCCCAGCAAGTCCTA  R: GAGAACCTTTTGCCGTGAAGC | 137 |
| *PROX1* | Prospero homeobox protein 1 | F: CCGTCCCTGGTCCAATTATCA  R: TCATGGTCAGGCATCACTGG | 115 |
| *PVALB* | Parvalbumin | F: GATGACAGACTTGCTCAACGC  R: GTGGTCGAAGGAGTCGACAG | 76 |
| *RPH3A* | Rabphilin 3A | F: CAGGGAAGAGAGGAGTTGGC  R: GATTGAAGGGGTCGGTCACT | 151 |
| *S100B* | S100 calcium-binding protein B | F: AGCTGTTTCCTTAGGTCGCA  R: GCTTTGTATTCACGGAGGCG | 128 |
| *SEMA6A* | Semaphorin 6A | F: GTAACTGCCTGCAGTATGTTGAA  R: CTTAAGGCTCGCCTCAACTG | 71 |
| *SLC12A2* | Solute carrier family 12 member 2 (NKCC1) | F: CGCCTGGTGTCAAGGATGT  R: TTGCCATCCTCTTCCTCATCTTTAT | 118 |
| *SLC12A5* | Solute carrier family 12 member 5 (KCC2) | F: TGGACCAAGGACAAATCGGT  R: CAAGTTCTCCCACTCCGGCT | 105 |
| *SLC17A6* | Solute carrier family 17 member 6 (vGLUT2) | F: AGGATTTTGGCCCCAGGAAA  R: GCTTCTTCTCTAGCACCCTGTA | 85 |
| *SLC17A7* | Solute carrier family 17 member 7 (vGLUT1) | F: CGCAGCTAACAGGGTTTTCG  R: TCCTCACGAAGATGACGCAG | 107 |
| *SV2B* | Synaptic vesicle glycoprotein 2B | F: CTGAATGTGGTTGCCCTGTCC  R: TCCCCATCCCTTCTTCCATCTA | 162 |
| *SYN1* | Synapsin 1 | F: AGCTGGCACAGAAACCTAGC  R: CAGAGACTGGGATTTGTTGAGC | 113 |
| *TTR* | Transthyretin | F: TGCTGGTGAATCCAAGTGTC  R: CACTTTCACGCCTACGTTCA | 85 |
| *VEGFA* | Vascular endothelial growth factor A | F: ACTGACACAGAACGACCCGT  R: AACGGCCATCCAATCCCAAG | 196 |

F = forward primer, R = reverse primer. ^¤^ primers for these genes were adopted from (Pan et al., 2012)
