# Supplementary material for: Insulin-like growth factor 1 supplementation supports motor coordination and affects myelination in preterm pigs
Source: Front Neurosci. 2023 Jun 19;17:1205819. doi: 10.3389/fnins.2023.1205819 (PMC10315495; doi:10.3389/fnins.2023.1205819)
Supplement: Supplementary file 3 [file Table_3.DOCX]

**Supplementary Table S3.** Effect of IGF-1 treatment on the expression of selected genes associated with neuronal maturation in caudate nucleus, cerebellum, and hippocampus as determined qPCR analysis.

| **Gene category** |  |  | **Caudate nucleus** | | | **Cerebellum** | | | **Hippocampus** | | |
| --- | --- | --- | --- | --- | --- | --- | --- | --- | --- | --- | --- |
| **Gene name** | **Gene symbol** | **Protein symbol** | **Fold change** | **SEM** | **p-value** | **Fold change** | **SEM** | **p-value** | **Fold change** | **SEM** | **p-value** |
| **IGF1 signaling** | | | | | | | | | | | |
| Insulin-like growth factor 1 | *IGF1* | IGF1 | 1.023 | 0.071 | 0.932 | 1.143 | 0.153 | 0.518 | 0.942 | 0.037 | 0.656 |
| Insulin-like growth factor 1 receptor | *IGF1R* | IGF1R | 1.007 | 0.109 | 0.648 | 0.883 | 0.096 | 0.836 | 0.877 | 0.033 | 0.189 |
| Insulin-like growth factor 2 | *IGF2* | IGF2 | - | - | - | 0.708 | 0.128 | 0.665 | - | - | - |
| Insulin-like growth factor 2 receptor | *IGF2R* | IGF2R | 0.980 | 0.040 | 0.383 | 0.982 | 0.066 | 0.778 | 1.057 | 0.063 | 0.684 |
| Insulin-like growth factor binding protein 3 | *IGFBP3* | IGFBP3 | 0.968 | 0.089 | 0.876 | 1.101 | 0.166 | 0.965 | - | - | - |
| Insulin receptor substrate 1 | *IRS1* | IRS1 | 0.924 | 0.046 | 0.159 | 1.175 | 0.154 | 0.679 | 0.935 | 0.059 | 0.502 |
| **Myelination** | | | | | | | | | | | |
| Myelin associated glycoprotein | *MAG* | MAG | 0.698 | 0.075 | 0.002** | 0.730 | 0.090 | 0.018* | 0.986 | 0.122 | 0.754 |
| Myelin basic protein | *MBP* | MBP | 0.619 | 0.047 | 0.002** | 0.773 | 0.076 | 0.085 | 1.078 | 0.119 | 0.788 |
| Myelin oligodendrocyte glycoprotein | *MOG* | MOG | 0.693 | 0.052 | 0.004** | 0.791 | 0.073 | 0.064 | 1.022 | 0.094 | 0.965 |
| Oligodendrocytic myelin paranodal and inner loop protein | *OPALIN* | OPALIN | 0.715 | 0.053 | 0.016* | 0.794 | 0.074 | 0.090 | 1.000 | 0.130 | 0.896 |
| **Differentiation and survival** | | | | | | | | | | | |
| Aldolase C | *ALDOC* | ALDOC | - | - | - | 1.086 | 0.073 | 0.519 | - | - | - |
| Ataxin 1 | *ATXN1* | ATXN1 | - | - | - | 1.075 | 0.067 | 0.399 | - | - | - |
| Caspase 3 | *CASP3* | CASP3 | 0.969 | 0.036 | 0.354 | 1.054 | 0.039 | 0.439 | - | - | - |
| Erythroblastic oncogene B 2 | *ERBB2* | ERBB2 | 1.015 | 0.064 | 0.530 | 0.953 | 0.048 | 0.635 | - | - | - |
| Hypoxia-inducible factor 1-alpha | *HIF1A* | HIF1A | 0.920 | 0.030 | 0.040* | 0.980 | 0.025 | 0.550 | 0.967 | 0.034 | 0.511 |
| Neurogenic differentiation factor 1 | *NEUROD1* | NeuroD1 | - | - | - | 0.961 | 0.036 | 0.467 | - | - | - |
| Neurogenic differentiation factor 2 | *NEUROD2* | NeuroD2 | 1.411 | 0.206 | 0.247 | 0.989 | 0.035 | 0.811 | - | - | - |
| Neurotrophic receptor kinase 2 | *NTRK2* | TrkB | 0.958 | 0.106 | 0.742 | 1.161 | 0.144 | 0.587 | - | - | - |
| S100 calcium-binding protein B | *S100B* | S100B | 0.887 | 0.041 | 0.052 | 0.875 | 0.051 | 0.028* | 0.957 | 0.069 | 0.684 |
| **Proliferation and migration** | | | | | | | | | | | |
| Cadherin 8 | *CDH8* | CDH8 | 0.899 | 0.068 | 0.076 | 0.922 | 0.055 | 0.449 | 1.054 | 0.046 | 0.662 |
| Eukaryotic translation elongation factor 1 alpha 1 | *EEF1A1* | EEF1A1 | 0.978 | 0.055 | 0.550 | 0.880 | 0.111 | 0.456 | 1.056 | 0.041 | 0.553 |
| Eukaryotic translation elongation factor 1 alpha 2 | *EEF1A2* | EEF1A2 | 1.051 | 0.060 | 0.628 | 0.936 | 0.106 | 0.337 | 1.072 | 0.063 | 0.905 |
| Ephrin A5 | *EFNA5* | EFNA5 | 0.965 | 0.058 | 0.500 | - | - | - | - | - | - |
| Basic fibroblast growth factor | *FGF2* | FGF2 | 0.929 | 0.033 | 0.248 | 1.069 | 0.043 | 0.445 | - | - | - |
| Growth associated protein 43 | *GAP43* | GAP43 | - | - | - |  |  |  | 1.111 | 0.089 | 0.589 |
| Megalencephalic leukoencephalopathy with subcortical cysts 1 | *MLC1* | MLC1 | 1.054 | 0.075 | 0.870 | 0.852 | 0.045 | 0.008** | - | - | - |
| Neuropilin 1 | *NRP1* | NRP1 | 1.021 | 0.044 | 0.657 | - | - | - | - | - | - |
| Semaphorin 6A | *SEMA6A* | SEMA6A | 0.944 | 0.043 | 0.141 | 0.997 | 0.056 | 0.994 | - | - | - |
| Vascular endothelial growth factor A | *VEGFA* | VEGFA | 0.931 | 0.046 | 0.332 | 1.000 | 0.058 | 0.828 | - | - | - |
| **Synaptic transmission** | | | | | | | | | | | |
| Cerebellin 1 precursor | *CBLN1* | CBLN1 | - | - | - | 0.891 | 0.043 | 0.160 | - | - | - |
| Dynamin 1 | *DNM1* | DNM1 | - | - | - | - | - | - | 1.114 | 0.050 | 0.213 |
| Protein piccolo | *PCLO* | PCLO | 0.918 | 0.041 | 0.281 | 1.076 | 0.046 | 0.448 | - | - | - |
| Rabphilin 3A | *RPH3A* | RPH3A | 0.941 | 0.047 | 0.520 | 0.991 | 0.038 | 0.406 | - | - | - |
| Solute carrier family 17 member 6 | *SLC17A6* | VGLUT2 | 0.870 | 0.082 | 0.348 | 1.119 | 0.124 | 0.359 | - | - | - |
| Solute carrier family 17 member 7 | *SLC17A7* | VGLUT1 | - | - | - | 0.910 | 0.054 | 0.198 | 1.038 | 0.045 | 0.850 |
| Synaptic vesicle glycoprotein 2B | *SV2B* | SV2B | 0.972 | 0.047 | 0.941 | 1.069 | 0.047 | 0.399 | - | - | - |
| Synapsin 1 | *SYN1* | SYN1 | - | - | - | - | - | - | 1.010 | 0.054 | 0.464 |
| **Dopamine system** | | | | | | | | | | | |
| Dopamine receptor D1 | *DRD1* | DRD1 | 1.035 | 0.062 | 0.982 | - | - | - | - | - | - |
| Dopamine receptor D2 | *DRD2* | DRD2 | 0.953 | 0.082 | 0.243 | 0.929 | 0.042 | 0.849 | - | - | - |
| **GABA system** | | | | | | | | | | | |
| Calbindin 1 | *CALB1* | CALB1 | 0.953 | 0.046 | 0.789 | 1.136 | 0.053 | 0.074 | - | - | - |
| Calbindin 2 | *CALB2* | CALB2 | 1.094 | 0.265 | 0.201 | 0.946 | 0.075 | 0.664 | - | - | - |
| Gamma-aminobutyric acid type A receptor subunit alpha 1 | *GABRA1* | GABRA1 | 0.870 | 0.109 | 0.198 | 1.074 | 0.034 | 0.295 | 1.024 | 0.082 | 0.770 |
| Gamma-aminobutyric acid type A receptor subunit alpha 3 | *GABRA3* | GABRA3 | 0.838 | 0.035 | 0.013* | 0.939 | 0.052 | 0.334 | 0.910 | 0.051 | 0.158 |
| Glutamate decarboxylase 1 | *GAD1* | GAD1 | 1.098 | 0.065 | 0.723 | 1.078 | 0.052 | 0.400 | - | - | - |
| Glutamate decarboxylase 2 | *GAD2* | GAD2 | 1.020 | 0.066 | 0.497 | 1.003 | 0.030 | 0.921 | - | - | - |
| Prospero homeobox protein 1 | *PROX1* | PROX1 | 0.890 | 0.060 | 0.136 | 0.998 | 0.037 | 0.930 | - | - | - |
| Parvalbumin | *PVALB* | PVALB | 0.925 | 0.089 | 0.369 | 0.943 | 0.036 | 0.399 | - | - | - |
| Solute carrier family 12 member 2 | *SLC12A2* | NKCC1 | 0.762 | 0.052 | 0.007** | 0.989 | 0.068 | 0.680 | 0.945 | 0.077 | 0.731 |
| Solute carrier family 12 member 5 | *SLC12A5* | KCC2 | 1.000 | 0.054 | 0.783 | 1.019 | 0.026 | 0.810 | 1.042 | 0.058 | 0.835 |
| **Glutamate system** | | | | | | | | | | | |
| Glutamate receptor 3 | *GRIA3* | GRIA3 | 0.939 | 0.052 | 0.784 | 1.098 | 0.039 | 0.161 | 0.998 | 0.055 | 0.878 |
| Glutamate receptor 4 | *GRIA4* | GRIA4 | 0.797 | 0.034 | 0.001** | 0.994 | 0.030 | 0.864 | 0.980 | 0.047 | 0.894 |
| **Serotonin** | | | | | | | | | | | |
| 5-Hydroxytryptamine receptor 2A | *HTR2A* | 5-HT2A | 0.933 | 0.060 | 0.460 | - | - | - | - | - | - |
| **Others** | | | | | | | | | | | |
| Aquaporin 4 | *AQP4* | AQP4 | 1.025 | 0.080 | 0.476 | 0.851 | 0.059 | 0.086 | - | - | - |
| Solute carrier family 2 member 3 | *SLC2A3* | GLUT3 | 1.050 | 0.043 | 0.180 | 0.991 | 0.100 | 0.872 | - | - | - |
| Transthyretin | *TTR* | TTR | - | - | - | - | - | - | 1.457 | 0.352 | 0.269 |
| Chondromodulin | *CNMD* | CNMD | - | - | - | - | - | - | 1.226 | 0.123 | 0.230 |

Significant differentially expressed genes are highlighted in light blue. * p < 0.05, ** p < 0.001.
