# Supplementary material for: Insulin-like growth factor 1 supplementation supports motor coordination and affects myelination in preterm pigs
Source: Front Neurosci. 2023 Jun 19;17:1205819. doi: 10.3389/fnins.2023.1205819 (PMC10315495; doi:10.3389/fnins.2023.1205819)
Supplement: Supplementary file 4 [file Table_4.DOCX]

|  |  | **Primary analyses** | | | | **Sensitivity analyses:**  **Birth weight ^a^** | | | **Sensitivity analyses:**  **Growth rate ^b^** | | |
| --- | --- | --- | --- | --- | --- | --- | --- | --- | --- | --- | --- |
| **Outcomes** | **Unit** | **Control** | | **IGF-1** | ***p*-value** | **Control** | **IGF-1** | ***p*-value** | **Control** | **IGF-1** | ***p*-value** |
| **Motor development** | | *n = 12-14* | | *n = 14-15* |  | *n = 10-11* | *n = 10-11* |  | *n = 8-9* | *n = 11-13* |  |
| Balance (mis-steps) – PND10 | Count | 3.000±0.278 | | 2.278±0.246 | 0.025* | 3.315±0.338 | 2.583±0.242 | 0.026* | 3.021±0.373 | 2.192±0.261 | 0.033* |
| Balance (mis-steps) – PND18 | Count | 1.764±0.186 | | 1.289±0.187 | 0.066 | 1.722±0.236 | 1.083±0.186 | 0.046* | 1.667±0.267 | 1.545±0.187 | 0.830 |
| Stride length – PND10 | Count | 1.081±0.027 | | 1.161±0.030 | 0.038* | 1.120±0.023 | 1.142±0.039 | 0.292 | 1.096±0.040 | 1.152±0.034 | 0.274 |
| Stride length – PND18 | Count | 1.048±0.023 | | 1.093±0.026 | 0.169 | 1.051±0.026 | 1.107±0.031 | 0.233 | 1.074±0.026 | 1.079±0.027 | 0.622 |
| **Cognitive development** | | *n = 11-12* | | *n = 12-16* |  | *n = 9-10* | *n = 8-11* |  | *n = 7-9* | *n = 10-12* |  |
| Operant conditioning | % | 72.73% | | 50.00% | 0.699 | 66.67% | 62.50% | 0.137 | 85.71% | 50.00% | 0.001*** |
| Novel object recognition | RI | 0.785±0.046 | | 0.705±0.065 | 0.566 | 0.794±0.039 | 0.761±0.067 | 0.341 | 0.734±0.047 | 0.683±0.076 | 0.904 |
| **Brain Growth** | | *n = 6-14* | *n = 10-16* | |  | *n = 4-10* | *n = 7-12* |  | *n = 4-9* | *n = 7-13* |  |
| MRI – white matter volume | cm^3^ | 1.241±0.070 | | 1.222±0.028 | 0.911 | 1.297±0.089 | 1.244±0.031 | 0.699 | 1.176±0.044 | 1.234±0.026 | 0.265 |
| MRI – grey matter volume | cm^3^ | 11.923±0.172 | | 11.842±0.191 | 0.795 | 12.112±0.164 | 11.979±0.216 | 0.659 | 11.782±0.239 | 11.695±0.192 | 0.552 |
| Relative total brain weight | % | 1.876±0.080 | | 2.023±0.102 | 0.696 | 1.782±0.060 | 1.852±0.056 | 0.877 | 1.843±0.066 | 2.064±0.121 | 0.284 |
| Relative caudate nucleus weight | % | 0.977±0.025 | | 0.879±0.026 | 0.003** | 0.967±0.034 | 0.857±0.027 | 0.007** | 1.017±0.025 | 0.897±0.030 | 0.007** |
| Protein synthesis rate – *in vivo* | Ks | 17.987±0.430 | | 20.150±0.609 | 0.013* | 17.764±0.554 | 20.299±0.740 | 0.046* | 18.599±0.279 | 19.883±0.679 | 0.179 |
| Protein synthesis rate – *ex vivo* | A.U. | 5642±1084 | | 13023±1950 | 0.002** | 5755±1320 | 14245±2287 | 0.001** | 5933±1385 | 14018±2450 | 0.004** |

**Supplementary table S4:** Sensitivity analysis to assess the effect of IGF-1 supplementation on brain development.

|  | | **Primary analyses** | | | **Sensitivity analyses:**  **Birth weight ^a^** | | | **Sensitivity analyses:**  **Growth rate ^b^** | | |
| --- | --- | --- | --- | --- | --- | --- | --- | --- | --- | --- |
| **Outcomes** | **Unit** | **Control** | **IGF-1** | ***p*-value** | **Control** | **IGF-1** | ***p*-value** | **Control** | **IGF-1** | ***p*-value** |
| **Immunohistochemistry - MBP** | | *n = 14* | *n = 16* |  | *n = 10* | *n = 12* |  | *n = 9* | *n = 13* |  |
| Periventricular white matter | Area% | 58.596±1.290 | 56.068±1.314 | 0.038* | 57.497±1.297 | 55.206±1.473 | 0.052 | 58.867±1.668 | 56.376±1.498 | 0.371 |
| Intracortical white matter | Area% | 48.559±0.715 | 45.392±0.867 | 0.009** | 48.294±0.782 | 45.034±1.028 | 0.031* | 48.713±0.875 | 45.236±0.997 | 0.034* |
| - Temporal | Area% | 52.596±0.955 | 49.345±1.241 | 0.052 | 52.112±0.973 | 49.005±1.617 | 0.114 | 52.741±1.032 | 48.715±1.417 | 0.073 |
| - Parietal | Area% | 46.670±0.764 | 43.418±0.952 | 0.022* | 47.034±0.765 | 42.922±1.013 | 0.008** | 46.848±0.942 | 43.601±1.118 | 0.097 |
| - Cingulate | Area% | 46.406±0.954 | 43.411±0.970 | 0.035* | 45.731±1.032 | 43.174±1.084 | 0.171 | 46.546±1.120 | 43.390±1.135 | 0.066 |
| **Gene Expression – Caudate nucleus** | | *n = 13* | *n = 15-16* |  | *n = 10* | *n = 9-10* |  | *n = 9* | *n = 11-12* |  |
| MPB | FC | 1.000±0.021 | 0.619±0.047 | 0.002** | 1.000±0.175 | 0.609±0.069 | 0.037* | 1.000±0.156 | 0.623±0.050 | 0.009** |
| MOG | FC | 1.000±0.088 | 0.649±0.065 | 0.004** | 1.000±0.118 | 0.671±0.105 | 0.089 | 1.000±0.092 | 0.623±0.074 | 0.006** |
| MAG | FC | 1.000±0.092 | 0.654±0.083 | 0.002** | 1.000±0.118 | 0.686±0.128 | 0.197 | 1.000±0.099 | 0.671±0.091 | 0.040* |
| OPALIN | FC | 1.000±0.102 | 0.715±0.053 | 0.016* | 1.000±0.129 | 0.736±0.074 | 0.084 | 1.000±0.110 | 0.708±0.059 | 0.017* |
| NKCC1:KCC2 ratio |  | 0.200±0.021 | 0.150±0.009 | 0.044* | 0.203±0.028 | 0.149±0.010 | 0.166 | 0.197±0.019 | 0.154±0.011 | 0.068 |
| **Gene Expression – PvWM** | | *n = 12-14* | *n = 12-16* |  | *n = 9-10* | *n = 9-12* |  | *n = 8-9* | *n = 9-13* |  |
| MBP | FC | 1.000±0.085 | 0.940±0.092 | 0.855 | 1.000±0.100 | 0.857±0.099 | 0.484 | 1.000±0.107 | 0.962±0.099 | 0.774 |
| MOG | FC | 1.000±0.100 | 0.964±0.104 | 0.870 | 1.000±0.109 | 0.840±0.083 | 0.457 | 1.000±0.127 | 1.010±0.120 | 0.998 |
| MAG | FC | 1.000±0.092 | 0.931±0.098 | 0.690 | 1.000±0.110 | 0.811±0.074 | 0.352 | 1.000±0.114 | 1.029±0.135 | 0.935 |
| OPALIN | FC | 1.000±0.098 | 1.018±0.133 | 0.809 | 1.000±0.097 | 0.903±0.125 | 0.603 | 1.000±0.126 | 1.033±0.148 | 0.815 |

Results presented as mean ± SEM, except results of operant conditioning test which are presented as percentage of animals reaching learning criteria. ^a^ Sensitivity analyses conducted by removing animals in the lowest 25^th^ percentile for birth weight. ^b^ Sensitivity analyses conducted by removing animals in the lowest 25^th^ percentile for growth rate (g/kg/day). RI – recognition index, MRI – magnetic resonance imaging, Ks - protein mass synthesized/day, A.U. – arbitrary unit, Area% - fraction of immunoreactive area, FC – fold change in relative expression compared to Control group, PvWM – periventricular white matter.
