# Supplementary figures and images for: Insulin-like growth factor 1 supplementation supports motor coordination and affects myelination in preterm pigs
Source: Front Neurosci. 2023 Jun 19;17:1205819. doi: 10.3389/fnins.2023.1205819 (PMC10315495; doi:10.3389/fnins.2023.1205819)

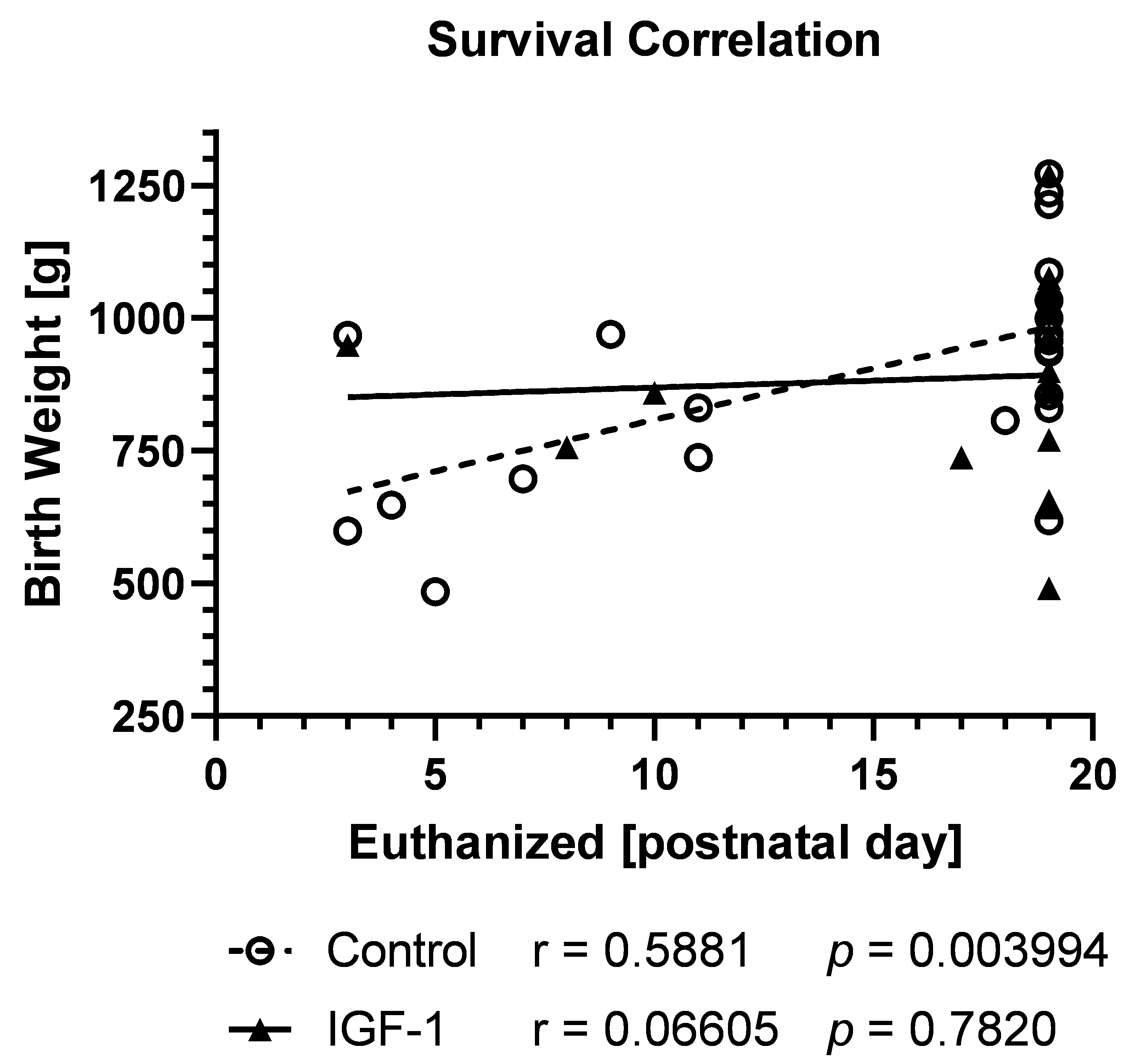

Supplement: Supplementary file 5 [file Image_1.TIF]

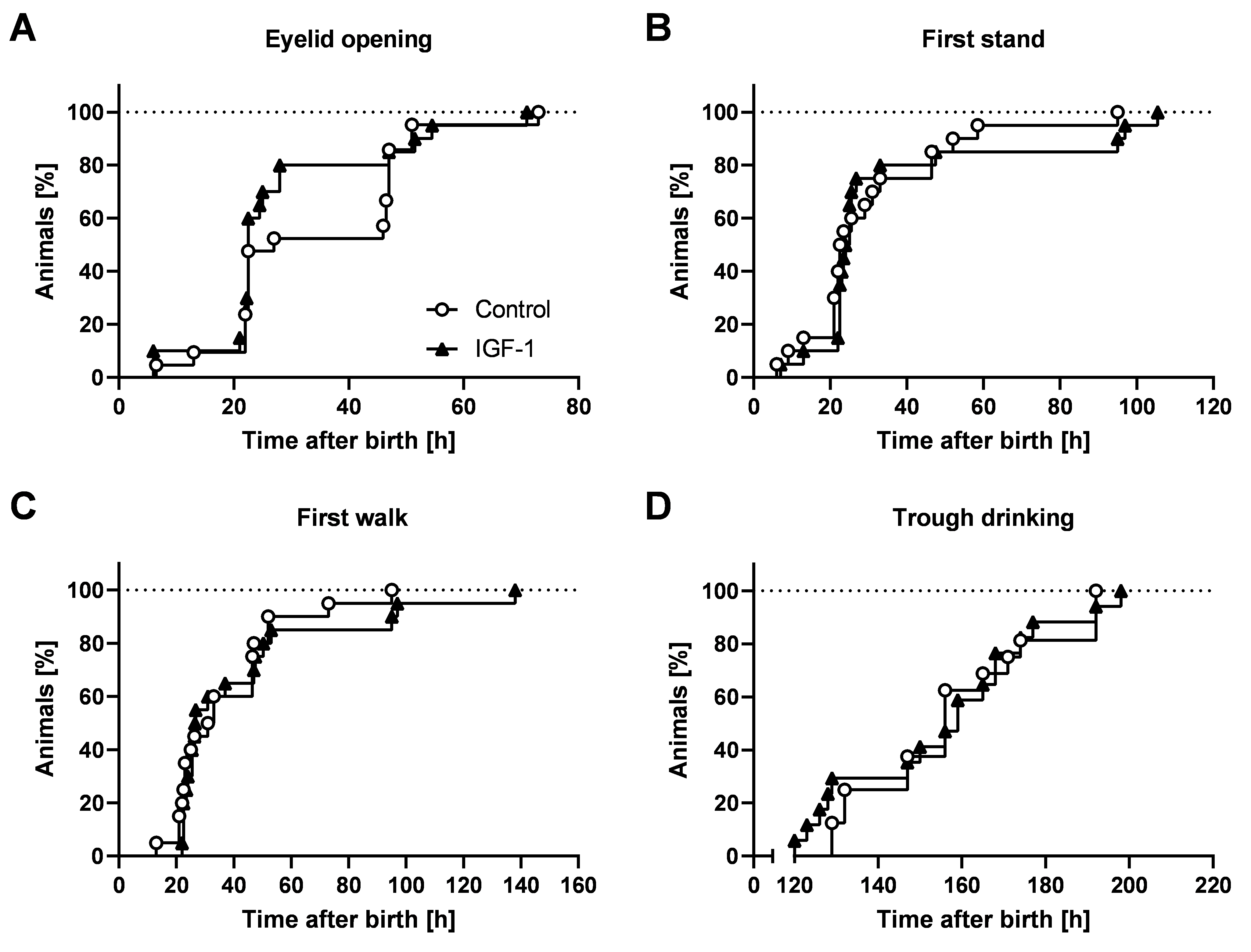

Supplement: Supplementary file 6 [file Image_2.TIF]

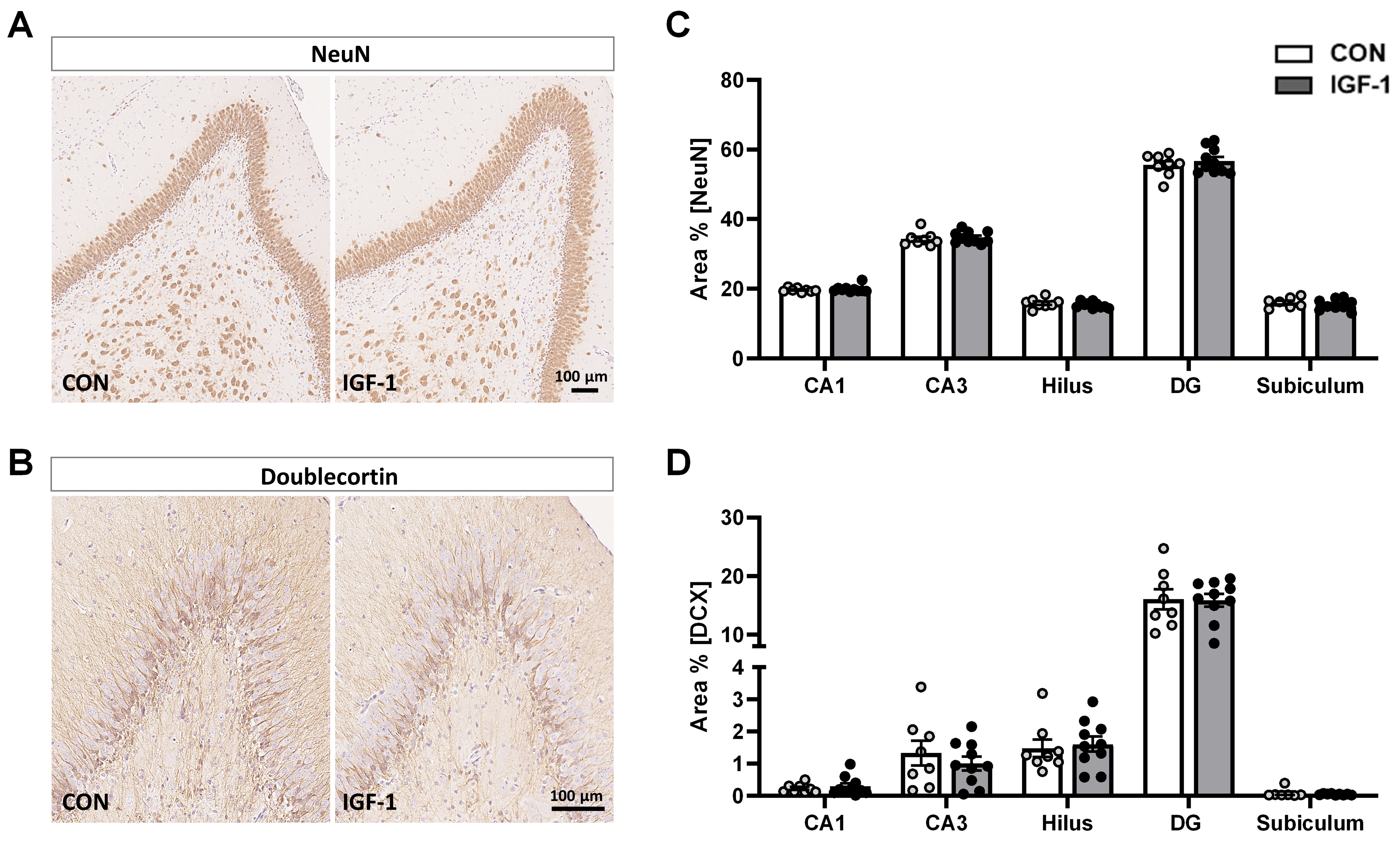

Supplement: Supplementary file 7 [file Image_3.TIF]
